# Evidence of balanced diversity at the chicken interleukin 4 receptor alpha chain locus

**DOI:** 10.1186/1471-2148-9-136

**Published:** 2009-06-15

**Authors:** Tim Downing, David J Lynn, Sarah Connell, Andrew T Lloyd, A K Bhuiyan, Pradeepa Silva, A N Naqvi, Rahamame Sanfo, Racine-Samba Sow, Baitsi Podisi, Olivier Hanotte, Cliona O'Farrelly, Daniel G Bradley

**Affiliations:** 1 Smurfit Institute of Genetics, Trinity College, University of Dublin, Dublin, Ireland; 2 Department of Molecular Biology and Biochemistry, Simon Fraser University, 8888 University Drive, Burnaby, British Columbia, V5A 1S6, Canada; 3 School of Biochemistry and Immunology, Trinity College, University of Dublin, Dublin, Ireland; 4 Department of Animal Breeding and Genetics, Bangladesh Agricultural University, Mymensingh, Bangladesh; 5 Department of Animal Science, University of Peradeniya, Peradeniya, Sri Lanka; 6 PARC, Animal Sciences Division, Islamabad, Pakistan; 7 INERA, Department of Agriculture and Environment, Ouagadougou, Burkina Faso; 8 Institut Sénégalais de Recherches Agricoles, Dakar, Senegal; 9 Department of Agricultural Research, CTA, Gaborone, Botswana; 10 International Livestock Research Institute (ILRI), PO Box 30709, Nairobi 00100, Kenya; 11 School of Biology, University of Nottingham, Nottingham, NG7 2RD, UK

## Abstract

**Background:**

The comparative analysis of genome sequences emerging for several avian species with the fully sequenced chicken genome enables the genome-wide investigation of selective processes in functionally important chicken genes. In particular, because of pathogenic challenges it is expected that genes involved in the chicken immune system are subject to particularly strong adaptive pressure. Signatures of selection detected by inter-species comparison may then be investigated at the population level in global chicken populations to highlight potentially relevant functional polymorphisms.

**Results:**

Comparative evolutionary analysis of chicken (*Gallus gallus*) and zebra finch (*Taeniopygia guttata*) genes identified interleukin 4 receptor alpha-chain (IL-4Rα), a key cytokine receptor as a candidate with a significant excess of substitutions at nonsynonymous sites, suggestive of adaptive evolution. Resequencing and detailed population genetic analysis of this gene in diverse village chickens from Asia and Africa, commercial broilers, and in outgroup species red jungle fowl (JF), grey JF, Ceylon JF, green JF, grey francolin and bamboo partridge, suggested elevated and balanced diversity across all populations at this gene, acting to preserve different high-frequency alleles at two nonsynonymous sites.

**Conclusion:**

Haplotype networks indicate that red JF is the primary contributor of diversity at chicken IL-4Rα: the signature of variation observed here may be due to the effects of domestication, admixture and introgression, which produce high diversity. However, this gene is a key cytokine-binding receptor in the immune system, so balancing selection related to the host response to pathogens cannot be excluded.

## Background

The chicken represents one of our most important sources of food protein worldwide but remains a potential threat to human health as a reservoir for diseases and food-borne pathogens. Emerging diseases such as avian influenza [1] provide a new impetus to investigate chicken immunity – in particular the relationship between population diversity and disease susceptibility.

The geographic distribution, population densities and disease epidemiology of chickens is likely to have changed dramatically since their domestication, undoubtedly shaping their genetic diversity. Novel diseases and increased incidence of infection would have challenged the chicken immune response, necessitating adaptive evolution at key immune genes. Evidence for such adaptation is found in the sequence conservation of immunity-related genes, the lowest of any functional category [2], and in several studies reporting the association of allelic variation at particular immune genes with susceptibility to infection. For example, different alleles at the chicken MHC-B locus are known to alter susceptibility to a diverse array of diseases [3]. Genes such as the chicken Mx gene, which determines susceptibility to the myxovirus [4], have been shown to be subject to selection [5,6]. Genes involved in the immune system therefore represent appealing candidates for examining the selective processes shaping genetic diversity. Knowledge about the nature of selection acting on a gene can illuminate their evolutionary history and can provide insight into the complex relationship between diseases and genes [7].

New large-scale sequencing projects in several avian species, for instance the zebra finch genome project http://songbirdgenome.org, now allow the genome-wide comparative analysis of avian genes and the detection of selection on a wider scale. Approximately 20% amino acid changes between chicken and zebra finch have been fixed by positive selection [8], so by comparing coding sequences (CDS) between these birds, chicken genes with signals suggestive of adaptation can be identified.

In this study, we report that the chicken interleukin receptor 4 alpha chain gene (IL-4Rα) showed a relative excess of nonsynonymous substitutions and may be subject to selection. It is associated with disease: for example, its expression is downregulated by the avian influenza virus during infection [1]. The human ortholog of this gene encodes a transmembrane receptor for IL-4 and IL-13, both of which are key immune system cytokines that initiate signalling pathways in the inflammatory response to infection [9]. The IL-4Rα gene was resequenced in 70 Asian and African village chickens, 20 commercial broilers, and in 6 closely related species: red, grey, Ceylon and green jungle fowl (JF), bamboo partridge and grey francolin. High allelic variation at this gene appeared to be balanced at two nonsynonymous SNP sites in particular. Although this may enhance immune system variability in response to challenges by pathogens, a consequence of the complex domestication history of the chicken is that introgression, multiple origins and migration are likely to have altered the pattern of diversity at this locus, complicating selection signatures.

## Methods

### Identifying candidate genes subject to selection

As the most extensively sequenced other bird species, all available zebra finch genes were compared with the chicken genome. This was achieved by clustering [10] validated zebra finch mRNAs and expressed sequence tags, then using chicken protein sequences to search this zebra finch database with Blastx, [11] and subsequently implementing T-Coffee [12] to generate 3,653 pairwise CDS alignments from the Blastx best-hit pairs (for details see supplementary methods).

Pairwise *d*_*N*_/*d*_*S *_(*ω*) was calculated for each CDS alignment using the codeml implementation of the PAML 3.15 package [13]. If synonymous and nonsynonymous mutations are neutral, the relative rates of each are expected to be equal so that *ω *= 1 [14]. Departures from this, where *ω *> 1 (*d*_*N *_>*d*_*S*_) suggest that nonsynonymous mutations are advantageous, and are maintained under directional selection. If *ω *< 1 (*d*_*N *_<*d*_*S*_) then the nonsynonymous SNPs may be deleterious since they are not preserved and are likely to be subject to purifying selection. We compared *ω *by maximum likelihood under two different models: a neutral model where *ω *was fixed = 1, and a model where *ω *was free to vary. These models were compared using a likelihood ratio test (LRT) to determine if the variable model was significantly better at explaining the data [13].

As a consequence of this conservative strategy of calculating *ω *across the entire gene length, genes may be discounted when the signal of directional selection is focused on specific regions or domains and thus obscured by purifying selection operating on the majority of the gene [15]. Many genes known to be subject to positive selection have 0.5 <*ω *< 1 [16], so using a lower cut-off point to identify candidate genes that may be subject to selection can be effective. Accordingly, chicken-zebra finch alignments with *ω *> 0.5 where the variable model was significantly favoured (p < 0.05) were identified. The annotation associated with the best human orthologs from the Panther database [17] was used to identify the function of chicken genes with relevance to the immune system.

The chicken IL-4Rα mRNA sequence (Refseq ID: XM_414885), initially determined by Boardman *et al*. (GenBank accession: CR407301) and Caldwell *et al*. [18], was aligned as a best hit to two clustered zebra finch ESTs, DQ213788 and DQ213787[19].

### Sample collection

A total of 90 chicken samples were acquired: 70 village birds from Asia and Africa (International Livestock Research Institute, Kenya) and 20 commercial broilers (Manor Farms, Co. Monaghan, Ireland). The commercial birds were composed of 10 Ross breed chickens from Ireland and 10 Hubbard Flex from France. The Asian and African samples included 10 chickens from each of 3 Asian (Bangladesh, Pakistan and Sri Lanka) and 4 African (Botswana, Burkina Faso, Kenya and Senegal) populations. One sample for each of six outgroup species were also sequenced. Three were species known to be closely related to the chicken: [20] Chinese bamboo partridge (*Bambusicola thoracica*, CAS89821), green JF (*Gallus varius*, CAS85707) and grey francolin (*Francolinus pondicerianus interpositus*, CAS87894) (Department of Ornithology and Mammalogy, Californian Academy of Sciences). And three other JF were also obtained: Grey (*Gallus sonneratii*), Ceylon (*Gallus lafayetii*) and red JF (*Gallus gallus*) samples (Wallslough Farm, Co. Kilkenny, Ireland). DNA was isolated from the samples using a phenol-chloroform extraction following a proteinase K digestion.

### Resequencing strategy

The UCSC http://genome.ucsc.edu, GenBank http://www.ncbi.nlm.nih.gov and Ensembl http://www.ensembl.org browsers were used to investigate the gene structure. At the time of analysis, a portion of the chicken IL-4Rα region was not displayed on these browsers, so the reference assembly (NC_006101) and reference contig (NW_001471454) were aligned with the IL-4Rα mRNA sequence from GenBank (XM_414885) using T-Coffee [12] to determine potential coding regions. A further T-Coffee alignment of the human and chicken IL-4Rα protein sequences identified chicken regions orthologous to variable regions in humans (Additional file 1): according to the Uniprot http://www.uniprot.org entry for human IL-4Rα (Uniprot: P24394), most variation is in the extracellular and cytoplasmic domains. Genscan was used to corroborate the predicted gene structure http://genes.mit.edu/GENSCAN.html.

**Figure 1:**
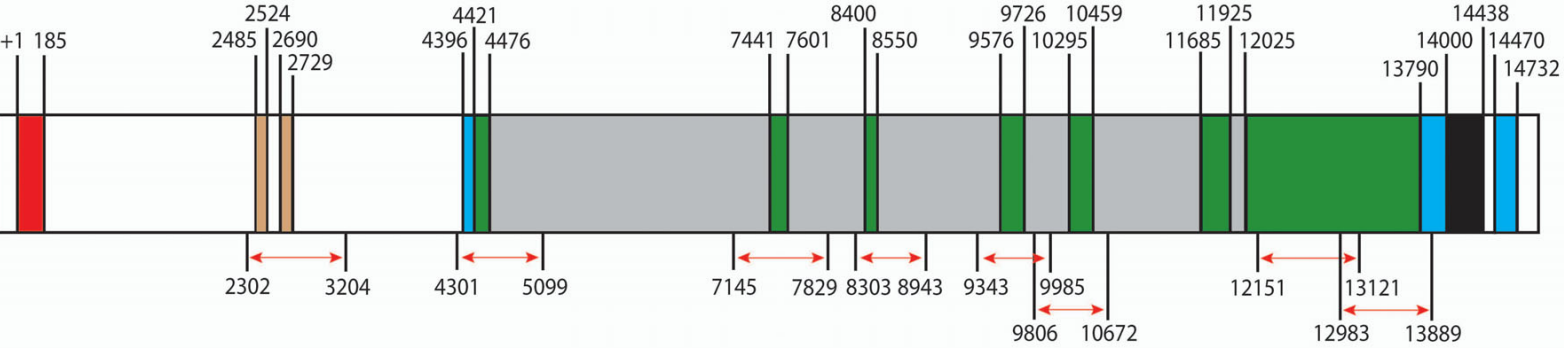
**Gene structure of IL-4Rα**. Exons are shown in green, introns in grey and amplicon regions by the red arrows. The UTRs are shown in blue, the leader sequence in red, unknown regions in black and promoter sequence is shown in beige. The numbers shown represent the base positions in relation to the GenBank entries for the mRNA and CDS.

PCR primers were designed using Primer3 software http://frodo.wi.mit.edu and were constructed by VHBio, UK http://www.vhbio.com. The details of the primer sequences and optimal parameters for their usage are available in Additional file 2 (Table S1). Each amplicon was amplified according to the PCR cycle setup (Table S2 in Additional file 2): 8 were successfully amplified for all 96 samples (Figure 1). The use of this large sample size increases probability of identification of a target of selection [21]. The forward and reverse PCR product sequences were determined by Agowa, Germany http://www.agowa.de.

### Sequence assembly

Sequencing reads were assembled into contigs using the Phred-Phrap-Consed-Polyphred pipeline programs http://www.phrap.org/phredphrapconsed.html Phrap v0.990319 and Phred v0.020425.c [22,23]. Bases were called with Consed [24] using P(base is correct) = 1–10^-S/10^, where S the base quality score [25]. Any bases with S < 20 were not included in the analysis, so all bases had at least a 99.0% probability of being correct: most had S ≥ 40 (99.99%). Only SNPs with high probability of being accurate (polyphred ranks 1, 2 or 3) were used in further analyses, and only SNPs in polyphred rank 1 were used for the outgroup samples. Polyphred version 5.0 [26,27] was used to assemble the data for further processing.

A list of the genotypes for each sample was collated and PHASE [28] was used to infer missing haplotypes. These assigned haplotypes were cross-referenced with haplotypes generated by Arlequin [29] to ensure consistency – both were identical. Any sequence sites with inadequate coverage across populations or continents, which had sub-standard base quality scores, or had insufficient coverage for either forward or reverse sequences, were removed – leaving a total of 5,298 bp for further analysis.

### Data analysis

DnaSP 4.0 [30,31] was used to analyse the polymorphic characteristics of the data and to perform a series of population genetic analyses. The numbers and types of SNPs were assessed. Nucleotide diversity was measured using *π*, the average number of nucleotide differences between sequences pairs [32]. The haplotype diversity (*Hd*, the number and frequency of haplotypes in the sample) [33], the number of haplotypes, Kelly's *Z*_*nS *_[34] and *θ*_*W *_= *4N*_*e*_*μ *[35] were determined. The four gamete test for the minimum number of recombination events (*R*_*M*_) [36] and *R *(the degree of recombination) [37] were calculated, as was the GC content.

A set of summary statistics were used to identify departures from neutrality using coalescent simulations: Fu and Li's *D *and *F *[38], Tajima's *D *[39], Fu's *F*_*s *_[40], Fay and Wu's *H *[41]. These tests were performed using DnaSP for 10^3 ^replicates with the following parameters estimated from the resequenced data: numbers of genotypes, segregating sites, total sites, sample sizes and rate of recombination. These simulations generated empirical distributions with which the statistical values were compared to determine the extent of their deviation from neutrality. It is an indication of non-neutral evolution if the observed values lie at the extremes of the distribution.

Median-joining haplotype networks were constructed using Network version 4.2.0.1 http://www.fluxus-technology.com. AMOVA tests [42] were conducted using Arlequin [29] with 10^3 ^permutations. Predictions to estimate the extent of functional impact for each radical substitution were conducted using PMut http://mmb2.pcb.ub.es:8080/PMut/[43].

The McDonald-Kreitman test [44] was implemented using DnaSP to examine the relative ratios of fixed and non-fixed nonsynonymous differences to fixed and non-fixed silent changes, which can indicate the presence of non-neutral evolution. Significance was based on a two-tailed Fisher's exact test.

### Selection at IL-4Rα among avian species

To investigate for evidence of selection in IL-4Rα between chicken and each of the 6 outgroups, CDS alignments were generated and *ω *was determined under a variety of models using codeml [13]. For this analysis, a chicken sequence from the most numerous haplotype was used (FJ542575). Although the chicken coding haplotypes observed at IL-4Rα were diverse, substituting this for other chicken genotypes yielded no significant changes to results, except at certain sites with model M8 for a divergent sample (FJ542675). The PAML models implemented here are sensitive to low numbers of sequences [45].

The free-ratio (M1) model was used to calculate tree branch lengths and *ω *for each species lineage in the sample. To identify specific codon sites with evidence of selection, site-specific models estimated *ω *for each site across the whole sequence by using a random sites model under Bayes empirical Bayes (BEB) [13,46,47]. For each model both the *ω *values and the fractions of sites affected are informative. For M1a, a neutral model, only two (K = 2) fixed *ω *values are permitted: *ω*_0 _= 0 (conserved) and *ω*_1 _= 1 (neutral). For M2a, a variable model, these two classes are used with an additional class (K = 3) where *ω *is freely estimated to allow for deviations from neutrality. Similarly, M7 is a neutral model that models K = 4 site classes sampled from a *β*-distribution, all of which have 0 ≤ *ω *≤ 1. Variable model M8 has the same four *β*-distributed classes as M7 with an additional class where *ω *> 1 (K = 5). A LRT was conducted between the paired neutral and variable models (M1a vs M2a, M7 vs M8). BEB was used to determine the posterior Bayesian probability of *ω *for each amino acid site: a significantly high posterior probability for this variable *ω *class suggests that a particular site is under selection, if *ω *> 1 and M8 (or M2a) is significantly favoured by the LRT [48]. Candidate positively selected sites from M8 were examined using PMut to assess the functional impact for each nonsynonymous substitution.

## Results

### Confirming the signature of selection at IL-4Rα

Tests on 3,653 chicken and zebra finch CDS pairwise alignments identified genes with *ω *> 0.5 where the variable model was significantly favoured from (Table S3 in Additional file 2; Additional file 3). From these, IL-4Rα was selected for further analysis because of its critical function in the immune response, including an implicated role in the anti-viral response [1]. Interestingly, another chicken immune gene identified by the pairwise comparison method (Progesterone-induced blocking factor) had a human ortholog that binds IL-4Rα.

IL-4Rα was resequenced in 7 closely related bird species: chicken, red JF, grey JF, Ceylon JF, green JF, grey francolin and bamboo partridge. An excess of nonsynonymous compared to synonymous substitutions was observed in all birds except red JF (Table S4 in Additional file 2). Branch-specific models of evolution, implemented in PAML [13] were used to investigate evidence of selection among the sequenced lineages. Using the free-ratio model, the branch leading to the *Gallus *genus was determined to have a high *ω *value (0.92) (Additional file 4), though this cannot be taken as strict evidence of positive selection. Consequently, site-specific models were implemented to investigate whether particular codon sites contributed to the evidence of selection. Model M8, one the most conservative models of site-specific evolution was determined to be significantly more favoured in comparison to the neutral M7 model (p = 5 × 10^-23^; Table 1). Bayes Empirical Bayes (BEB) was used to estimate the proportion of sites under positive selection: 48 (9.8%) of the sites had *ω *> 9.5, values much greater than that expected under neutrality [47]. Under M8, 28 sites were identified with a BEB posterior probability of at least 95% for *ω *> 1 (Table 2). There were substitutions between the chicken and red JF sample or genome sequence at 6 of these sites (5, 517, 547, 590, 628 and 665). PMut found 4 substitutions at these sites would have a neutral effect on protein structure (Table S5 in Additional file 2).

**Table 1:** Generated PAML parameters for free-ratio (M1) and significant site-specific test (M2a, M1a; M7, M8) results for IL-4Rα.

Model	Parameters	Likelihood	*ω *= *d*_*N*_/*d*_*S*_	2*ΔML*	P value
M1	*ω *= estimated independently for all	-2907.434066	See additional file 4	-	-

M2a	*ω*_0 _= 0 (90.25%)	-2798.740466	*ω*_2 _= 10.30236 (9.75%)	102.747134	4.88 × 10^-23^
M1a	*ω*_0 _= 0 (80.13%)	-2850.114033	*ω*_1 _= 1 (19.87%)		

M8^1^	*ω*_0–9 _< 0.08 (9.03% each)	-2798.740987	*ω*_10 _= 10.30383 (9.75%)	102.747656	4.88 × 10^-23^
M7	*ω*_0–7 _= 0 (10.0% each)	-2850.114815	*ω*_8,9 _= 1 (10.0%)		

^1 ^Bayes Empirical Bayes analysis suggests 28 sites where P(*ω *> 1) > 95.0%. *2ΔML *is twice the difference of the likelihoods of the variable minus the neutral model. The number of degrees of freedom is 2 for the site-specific model LRTs.

**Table 2:** Sites potentially under selection according to BEB analysis of PAML M8 results for the most frequent haplotype.

Base position	P	aA	*ω *value	S.E.	P(*ω *> 1)	Exon	Bases	SNP alleles and amino acids
4429–31	3	T	9.983	0.878	0.998	1	ACA	V, F: CCA (P); B: GCA (A)
4435–37	5	F	10.002	0.772	1.000	1	TTT	Chicken, C: CTT (L); R, V, F: TTC (F); B TTG (F)
4477–79	19	L	9.996	0.807	0.999	1	CTG	V, F: CGC (R); B: CCA (P)
7453–56	23	V	9.951	1.029	0.995	2	GTT	V, F: TTT (F); B: CTT (L)
7534–36	50	E	10.003	0.77	1.000	2	GAA	V, F: CCA (P); B: CGA (R)
7582–84	66	L	10.000	0.786	1.000	2	CTT	V, F: TTT (F); B: AAT (N)
7594–5, 8394	70	R	9.984	0.871	0.998	2, 3	AGA	V: TCA (S); F: ATA (M)
9583–85	125	T	9.999	0.788	1.000	4	ACT	C, B: GCT (A); V, F: TCT (S)
9631–33	141	L	9.771	1.636	0.976	4	TTG	C, G, B: CTG (L); V, F: ATG (M)
9646–48	146	S	9.995	0.811	0.999	4	AGC	V, F: CGC (R); B: GGC (G)
9715–17	169	Q	9.972	0.933	0.997	4	CAA	V, F: CGC (R); B: CCC (P)
9721–23	171	E	9.970	0.942	0.997	4	GAA	V, F: GCA (A); B: GGA (G)
12367–69	418	M	9.661	1.904	0.964	9	ATG	V: CTG (L); B: GTG (V); F: TTG (L)
12628–30	509	A	9.895	1.253	0.989	9	GCA	V, F: GTA (V)
12631–33	510	R	9.966	0.963	0.996	9	AGA	V: AGT (S); B: AGG (R); F: AGC (S)
12652–54	517	H	9.995	0.811	0.999	9	CAC	Chicken, R: CAT (H); RJF, F, V: CAA (Q); B: AAC (N)
12661–63^	520	P	9.110	2.54	0.930	9	CCT	Chicken, R, RJF: CTT (L)
12742–44 *	547	I	9.619	2.097	0.954	9	ATA	R, C: TTA (L)
12823–25	574	H	9.941	1.07	0.994	9	CAT	V, F: CAC (H); B, F: CAT (H)
12844–46	581	V	9.976	0.914	0.997	9	GTG	V, F: ATG (M); B: CTG (L)
12871–73	590	G	9.580	2.076	0.956	9	GGC	Chicken, RJF, B, F, V: AGC (S)
12955–57 *	618	E	9.622	2.092	0.955	9	GAG	V, F, B: GCG (A)
12979–81	626	S	9.579	2.078	0.956	9	AGC	V: CGC (R); F: GGC (G)
12985–87	628	E	9.934	1.101	0.993	9	GAA	Chicken: GAG (E); RJF, R, G, C: GAC (D)
13042–44	647	A	9.661	1.902	0.964	9	GCC	V, B, F: GTC (V)
13078–80	659	N	9.727	1.746	0.971	9	AAT	V, F: AAA (K); B: AAC (N)
13096–98	665	R	9.981	0.889	0.998	9	CGA	Chicken: CAA (Q), TGA (stop); R, G, C: AGA (R): RJF, F: ATA (M); V: ACA (T); B: AAA (K)
13123–25	674	S	9.950	1.033	0.994	9	TCT	V: TGT (C); B: TTT (F); F: TAT (Y)
13138–40	679	A	9.994	0.82	0.999	9	GCA	V: GGC (G); B: GTG (V); F: GGT (G)

P is the amino acid position. S.E. is the standard error for *ω*. B stands for bamboo partridge, F for grey francolin, V for green JF, C for Ceylon JF, R for red JF, G for grey JF and RJF for the genome sequence. * Significant in analysis with divergent sample FJ542675 only. ^Almost significant.

### SNP and Population diversity

Of the 100 SNPs observed among the chicken populations, 7 were singletons. In protein-coding regions 17 SNPs were observed: 10 were nonsynonymous and 7 were synonymous. Assuming red JF was the primary ancestral origin of diversity at this gene, some replacement mutations between red JF and chicken are potentially associated with the domestication process. In the chicken 7 nonsynonymous substitutions were identified as segregating at high frequencies (55% or more): F5L, L520P, S590G, L594R, M665R, S670Y and T692S (Table 3, Additional file 5).

**Table 3:** Frequencies and predicted functional impacts of chicken nonsynonymous SNPs on the IL-4Rα protein product compared to the red JF genome sequence

Base Positions	Amino Acid			Prediction	Score	Certainty	N^2^	Outcome
	Position	Red JF	M.A.					
4435–37	5	F	L	neutral	0.316	3	111	not significant
4450–52	10	T	A	neutral	0.104	7	1	neutral
9622–24	138	N	H	neutral	0.320	3	1	not significant
12661–63	520	L	P	neutral	0.413	1	102	not significant
12871–73	590	S	G	neutral	0.329	3	173	not significant
12883–85	594	L	R	neutral	0.270	4	174	not significant
13096–98	665	M	R	neutral	0.495	0	172	not significant
13096–98	665	M	Q	neutral	0.119	7	7	neutral
13096–98	665^1^	R	Q	neutral	0.510	5	7	not significant
13096–98	665^1^	R	stop	-	-	-	1	deleterious
13111–13	670	S	Y	neutral	0.036	9	163	neutral
13111–13	670	S	F	neutral	0.061	8	17	neutral
13111–13	670^1^	Y	F	neutral	0.023	9	17	neutral
13177–79	692	T	S	neutral	0.037	9	157	neutral
13177–79	692	T	N	neutral	0.060	8	23	neutral
13177–79	692^1^	S	N	neutral	0.028	9	23	neutral

M.A. is the minor allele(s), in some cases this is the most frequent in the chicken samples. Amino acid sites 5, 10, 524 and 669 are polymorphic in the outgroup samples as well. ^1 ^Polymorphic within chicken samples only. ^2 ^N is the number of observed samples with the M.A. Substitutions where the PMut certainty values ≤ 6 did not have statistical support for the predicted change.

**Figure 2:**
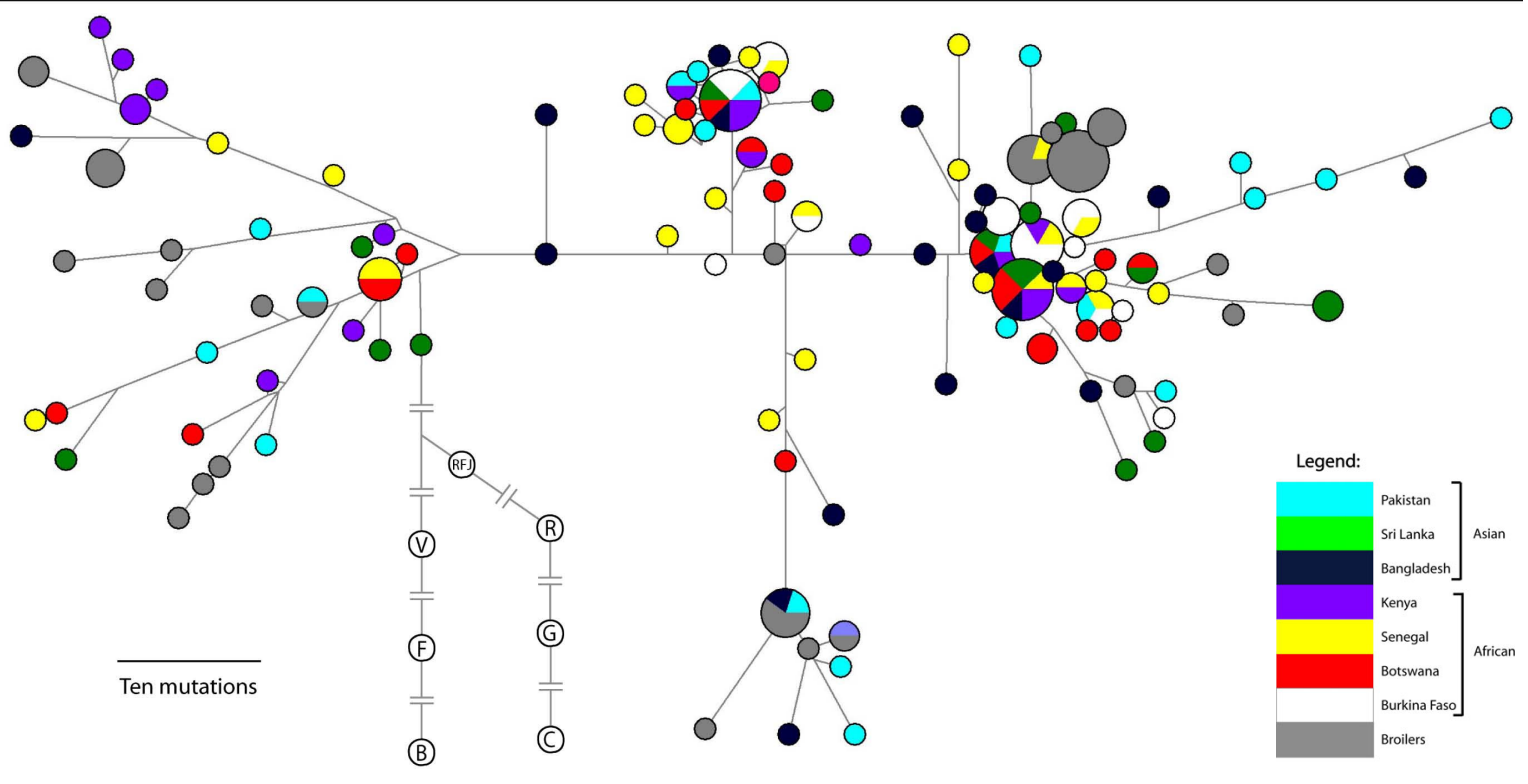
**Median-joining network of chicken haplotypes for all SNPs**. Populations are denoted in the legend. Branch lengths are proportional to the number of mutational differences between haplotypes. The outgroup samples are represented by the colourless nodes. Branch lengths are considerably reduced in order to show the details of the chicken population network. V represents the green JF sequences; F the grey francolin; B the bamboo partridge; G the grey JF; C the Ceylon JF; R the red JF; and RJF the genome sequence.

**Figure 3:**
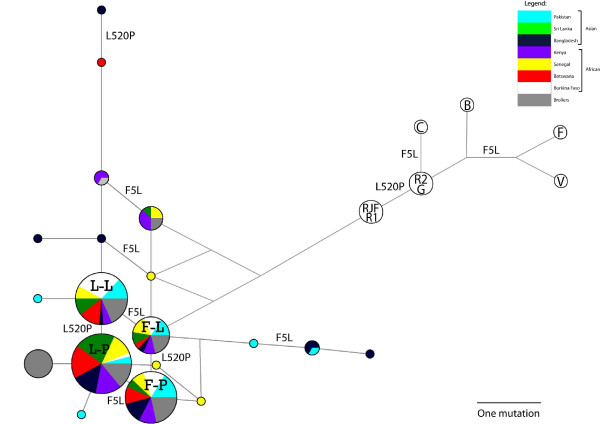
**Median-joining network of chicken haplotypes for nonsynonymous SNPs**. Populations are denoted in the legend. Branch lengths are proportional to the number of mutational differences between haplotypes. The outgroup samples are represented by the colourless nodes. V represents the green JF sequences; F the grey francolin; B the bamboo partridge; G the grey JF; C the Ceylon JF; R1 and R2 the red JF; and RJF the genome sequence.

The generation of median-joining networks (Figure 2) illustrated a high degree of allele diversity among samples and little geographical structuring among populations. The number of genetically divergent high-frequency haplotypes showed a trend of balanced diversity (Figure 2). When only the nonsynonymous SNPs were examined, an interesting pattern of dominant haplotypes emerged (Figure 3); when silent SNPs were included, recombination obfuscated these groups (Additional files 6 &7). Four haplotypes containing 81% of the 180 genotypes were characterised by substitutions at two sites: F5L and L520P. The 4 alleles possible at these 2 sites (F-L, F-P, L-L and L-P) were present in all 8 populations. No single variant was dominant among the samples: 32 were F-L, 38 were F-P, 46 were L-L and 64 were L-P. Both sites 5 and 520 showed evidence for positive selection in the site-specific test in codeml (Table 2, Additional file 8). Here, red JF and chicken both shared L520 and P520 alleles as well as F5, but L5 was unique to chicken.

The feature of high population diversity and low geographic partitioning in the networks was apparent in the analysis of variation using AMOVA with the Arlequin package [29]. This assessed the extent of partitioning of diversity at different levels of population structure. Most variation lay within the populations (94.1%, p < 1 × 10^-5^), a trend seen in other studies of chicken populations [48,49]; the remainder partitioned between the populations (1.8%, p = 0.060) and the continents (4.1%, p = 0.033).

### Summary statistics and tests of neutrality

There was further evidence for the trend of elevated allelic diversity: 115 haplotypes were observed in just 180 genotypes. This was reflected in the high *Hd *value, a statistical measure of haplotype diversity (Table 4). Fu's *F*_*S *_was highly negative, signifying an excess of rare alleles. Nucleotide, haplotype and SNP diversity were all higher in Asia than in Africa as expected, despite sampling fewer birds in Asia (30) than in Africa (40).

**Table 4:** SNP data, summary statistics and tests of neutrality

Test	N^1^	S^2^	H^3^	*Hd*^4^	*Π*^5^	*θ*_*w*_^6^	Tajima's *D*	Fu & Li's		Fay & Wu's *H*	Fu's *F*_*s*_
								*D*	*F*		
All	90	100	115	0.990	5.19	3.37	1.69	1.22	1.86	-21.40	-34.06
*P value*			<*0.001*	*<0.001*	*ns*	*0.001*	*<0.001*	*0.027*	*<0.001*	*0.010*	*0.015*
Asia	30	95	51	0.993	5.37	3.89	1.32	1.25	1.68	-19.45	-16.06
*P value*			*<0.001*	*0.007*	*ns*	*0.005*	*<0.001*	*<0.001*	*0.009*	*0.008*	*0.012*
Africa	40	86	53	0.983	4.72	3.36	1.36	1.11	1.55	-27.34	-10.13
*P value*			*ns*	*ns*	*ns*	*0.002*	*0.001*	*0.031*	*0.002*	*0.002*	*ns*
Broilers	20	79	23	0.944	5.14	3.51	1.69	2.15	2.47	-10.85	1.38
*P value*			*0.010*	*0.003*	*ns*	*0.018*	*<0.001*	*<0.001*	*<0.001*	*ns*	*0.007*

^1 ^Chickens sampled. ^2 ^SNPs. ^3 ^Haplotypes. ^4 ^Haplotype diversity. ^5 ^Mean pairwise differences per kb. ^6 ^Watterson's estimator per kb. Only p values generated by simulations < 0.05 are given; p > 0.05 are denoted "ns". The resequenced region length is 5,298 bp.

The significantly positive Tajima's *D *in Asia and Africa (Table 4) and in each of their populations (Table S6 in Additional file 2) was paralleled by a highly negative Fay and Wu's *H*, an indicator of an excess of derived alleles. Together, these metrics suggest a clear tendency for alleles to rise to mid- or high- frequency levels. Tests on the protein-coding portion of the gene alone indicated a significantly negative Fay and Wu's *H *(-3.02, p = 0.04) and a less positive Tajima's D (0.61); the latter may be a consequence of stronger conservation in coding regions, which appears to limit diversity, except at sites 5 and 520.

Moderate recombination was detected at IL-4Rα: for the given value of the recombination rate *R*, coalescent simulations showed the minimum number of recombination events (*R*_*M*_) was significantly high among all groups (Table 5).

**Table 5:** Recombination at IL-4Rα according the percentage GC content, Hudson's *R *and *R*_*M *_and Kelly's *Z*_*nS *_per kb from DnaSP

GC content (%)				*R*_*M*_^1^				*Z*_*nS*_^2^
Total	Coding	Non-coding	*R*	All	Asia	Africa	Broilers	
44.5	46.3	43.8	33.60	35	27	21	17	66.13

^1 ^Coalescent p < 0.001 for all, p < 0.001 for Asia, p = 0.004 for Africa and p = 0.013 for broilers. ^2 ^Coalescent p = 0.017.

Evidence of non-neutral evolution was evident from the McDonald-Kreitman [44] test results. The McDonald-Kreitman test examines the relative ratios of fixed and non-fixed nonsynonymous differences to fixed and non-fixed silent changes between species. Purifying selection may explain a rate of fixation of nonsynonymous differences much lower than that for silent substitutions. Alternatively, if there is a significant excess of fixation of nonsynonymous changes compared to silent ones, then directional selection may be present. The chicken genotypes were tested against the red JF genome sequence and also against each of the outgroup samples. Both tests showed an excess of fixed nonsynonymous substitutions (p = 0.002 with the genome sequence, p = 0.040 with all the outgroup samples; Table 6), indicating that selection may have affected the evolution of this gene.

**Table 6:** McDonald-Kreitman tests between the chicken populations and the red JF genome sequence and the outgroup samples.

Test	Type	Intraspecific	Interspecific	P value
Chicken & genome sequence	Silent	6	93	0.0016
	Nonsynonymous	6	10	

Chicken & 6 outgroups	Silent	6	447	0.0403
	Nonsynonymous	4	71	

## Discussion

### Identifying IL-4Rα as a candidate for resequencing

A pairwise comparison of *ω *= *d*_*N*_/*d*_*S *_in chicken and zebra finch genes identified IL-4Rα as having an elevated rate of nonsynonymous substitutions, suggesting it as a candidate for positive selection [50], though relaxed selective constraint has been observed in other domestic species [51]. Due to an important role in the host immune response and evidence of selection in humans, IL-4Rα was resequenced in 6 closely related birds and subsequently in 70 global village chickens and 20 commercial broilers. An analysis of sequence data from these 6 related species identified a large number of sites likely to be subject to positive selection, supporting the initial detection of IL-4Rα as a candidate gene undergoing adaptive evolution. Probable confounding factors in these results, however, are the complex domestication history of these populations and high rate of recombination identified at this locus.

The identification of chicken IL-4Rα is of particular interest given the vital role played by its human ortholog as a regulator of IgE production and T_*H*_2 cell differentiation [52,53]. The critical role of human IL-4Rα in the immune response is evidenced by its differential expression during particular infections and its association between polymorphism and disease susceptibility; it facilitates gastrointestinal nematode clearance [54] and its expression is upregulated in response to HIV-1 infection [55]. Variation in human IL-4Rα has been shown to affect signal transduction [56] and to modulate T_*H*_1/T_*H*_2 balance in the blood [57], as well as contributing to various allergies [9] and to mumps virus infection susceptibility [58]. Selection at IL-4Rα in human populations may be driven by different T_*H*_1 (viral and bacterial) and T_*H*_2 (parasitical) immune responses to pathogens [52], and the dysregulation of such components of immunity may be associated with atopy [59].

### The origin of diversity at IL-4Rα

Although nucleotide diversity at this gene (5.19 per kb) was comparable to that observed between red JF and domestic fowl (5.36 per kb on average) [2], the substantial excess of haplotypes was suggestive of non-neutral evolution. Despite this, the significantly positive Fu and Li's *D *and *F *values show that there was a relative deficit of singletons [39]. A deficit of rare alleles in commercial chicken lines has been observed in other studies comparing wild and standard breeds [65]. In this study, the *Hd *and Fu's *F*_*S *_values highlighted this rare allele deficiency in the commercial broilers, in contrast with the excess of haplotypes in the Asian and African samples. In addition, the significantly high *R*_*M *_values indicated that some recombinant alleles were present in the populations, implying either relaxed selective constraint or adaptive processes favouring allelic diversity.

Tajima's *D *compares the proportions of low- to medium-frequency alleles and is an indicator of directional selection when negative, and balancing selection when positive [40] (Tajima 1989). Fay and Wu's *H *measures the relative frequency of derived alleles, which increases when there are more high-frequency haplotypes [42]. The observed surplus of mid- and high-frequency haplotypes at the IL-4Rα locus has generated highly significant *D *and *H *values that are more extreme than those observed by other studies of disease-associated chicken genes [6,48] – however, *D *and *H *are likely to be affected by demographic aspects of chicken history and the samples pooling [66].

The networks were diffused into several divergent high-frequency haplotype clusters with high intra-population diversity. A distinctive set of balanced alleles was apparent when silent substitutions were removed. The signal of balanced diversity in the chicken populations appeared to centre around two nonsynonymous substitutions: F5L and L520P. All four variants at these two sites were segregating in the 8 populations surveyed at similar frequencies. Site-specific models of evolution identified both these sites as likely subject to selection across species.

An alignment of the chicken and human IL-4Rα protein sequences identified the amino acid positions orthologous to sites 5 and 520 in chicken (Additional file 1). The site orthologous to the latter is segregating in humans (C431R, rs1805012) [67,68] at an intermediate frequency of over 10% in the population [69], similar to the chicken polymorphism. Substitution C431R is in the cytoplasmic domain of the receptor and is linked with better survival from gliomas in humans [70]. The human amino acid position orthologous to chicken site 5 is conserved (F10) and is located in the signal peptide of the protein, indicating that the L5 chicken variant might affect activation of the receptor protein.

There is series of shared population genetic properties between chicken and human IL-4Rα that may be the result of equivalent functional roles for each. The genes possess comparable McDonald-Kreitman test results and positive Tajima's *D *values [49] and share orthologous high-frequency nonsynonymous SNPs (L520P and C431R). And given that several amino acid substitutions in IL-4Rα affect disease susceptibility in humans (see Franjkovic *et al*. [71]), the variability at nonsynonymous substitution sites in chickens is likely to be of biological importance.

The balanced and elevated variation and possible selective processes at chicken IL-4Rα may be in response to the common pathogens and the range of pleiotropic roles that the receptor plays in facilitating cytokine binding in the innate immune response. The trend of high diversity fuelled by balancing selection is seen in other chicken immune genes including MHC-B [3], Mx [6] and IL1B [48], which initially suggests that immune system genes may maintain high diversity in order to respond to a wide array of pathogens.

Another explanation for the observed elevated balanced diversity is that multiple domestications of red JF and genetic introgressions of other JF have both enhanced and distorted variation at this locus. The lack of observed geographic structure, which has also been observed at other chicken genes, [48] may be in part a consequence of this. There are likely to have been multiple events of chicken domestication in South and South-East Asia [72-74]. And though the red JF is the main source of chicken genetic diversity [2,75], genetic introgressions have come from other wild JF [76]: possibly from Ceylon JF [77] and unambiguously at the yellow skin locus from grey JF [78]. Wild red JF and domestic village strains are closely related [50,79], indicating that introgressions of red JF may have continued after domestication. Here, networks of IL-4Rα indicate that red JF is the most closely related wild relative to the domestic chicken. This does not exclude the possibility of multiple contributions of different genetic sources of JF. If admixture of different sources occurred sufficiently early through trading and migration [80,81] this may explain the presence of the four alleles at the two nonsynonymous sites in each population. Regardless of whether this signal of high and balanced diversity is from biological pleiotropy or from multiple origins, it is persisting, indicating that it may have an important role in current chicken immunity.

## Conclusion

This study shows evidence for high and balanced diversity at the chicken IL-4Rα gene, which was initially identified through the evaluation of the rate of nonsynonymous to synonymous substitutions in pairwise comparisons of chicken and zebra finch orthologs. This strategy incorporated functional and literature information to detect a suitable gene for resequencing in African, Asian and commercial chicken samples, as well as in related JF and bird species. Haplotype networks, tests of neutrality and summary statistics indicated a signal of balanced nonsynonymous polymorphisms at two sites in the IL-4Rα gene. Networks showed that red JF is the primary source of diversity at this gene. The elevated and balanced diversity present in all the populations might be a result of the chicken's history of multiple domestications [72-74], introgressions [76-78] and subsequent admixture of different types [79-81]. However, the identification of two potentially functionally significant SNPs as fulcrums of the balancing signal suggest that the functions of IL-4Rα in the immune system may affected by selective processes for specific allelic variants in response to new pathogenic challenges during domestication.

## Abbreviations

CDS: coding sequence; IL-4Rα: interleukin-4 receptor alpha-chain gene; JF: jungle fowl; LRT: likelihood ratio test; π: nucleotide diversity; *ω*: the rate of nonsynonymous mutations per nonsynonymous site (*d*_*N*_) divided by the rate of synonymous mutations per synonymous site (*d*_*S*_); UCSC: University of California Santa Cruz.

## Authors' contributions

DB, COF, AL and DL designed the study. TD and DL completed the bioinformatic gene identification. OH, TD, AB, PS, AN, RS, RSS and BP carried out sample collection. TD and SC prepared the samples. TD did the resequencing, data assembly and conducted population genetic tests. TD, DL, DB, COF and AL wrote the manuscript. All authors read and approved the final manuscript.

## Supplementary Material

Additional file 1 **An alignment of chicken and human IL-4Rα protein sequences**. The consensus human IL-4Rα sequence isoform a (GenBank accession number NP_000409) and the consensus chicken sequence (XP_414885) were aligned with T-Coffee [12]. The sites marked green were subsequently found to be candidates for selection according to PAML M8 BEB results. Sites marked green and in red letters indicate those subsequently observed as segregating in chicken populations and/or with differences between the chicken and the red JF sequences. Click here for file

Additional file 2 **Supplementary Methods and Results**. A file containing details of supplementary methods and results implemented, including tables, detailing: the identification of putative chicken-zebra finch orthologous alignments, PCR and primer sequences, resequencing details, alignment parameters, methodology for pairwise comparisons of chicken and zebra finch genes, and the details of chicken genes identified that interact with IL-4Rα. Click here for file

Additional file 3 **The numbers of genes (N) in classes of *ω *values from pairwise alignments of chicken-zebra finch gene sets where the variable model was favoured (p < 0.05)**. The y-axis is on a logarithmic scale. The *ω *values on the x-axis are classes into groups of 0.01, with the exception of values greater than 1, which are classed as 0.99–1.00. Click here for file

Additional file 4 **Codeml neighbour-joining phylogeny of IL-4Rα**. Branch lengths were estimated by maximum likelihood under the free-ratio model, which assumes an independent *ω*-ratio for each branch: these values are displayed. The branch length displayed is 0.1 of the total branch lengths for the tree. The *ω *for chicken was 0.4181 when sample FJ542675 was used instead of FJ542575. The *ω *values for grey and Ceylon JF are high because no synonymous SNPs were observed. Click here for file

Additional file 5 **Genotypes at SNP sites polymorphic in the chicken for all samples**. The coding sites are marked as "Y" if nonsynonymous. Samples are from Pakistan (FJ542565-FJ542584), Burkina Faso (FJ542585-FJ542604), Senegal (FJ542605-FJ542624), Sri Lanka (FJ542625-FJ542644), Botswana (FJ542645-FJ542664), Bangladesh (FJ542665-FJ542684), Kenya (FJ542685-FJ542704), Broilers (FJ542705-FJ542744), bamboo partridge (FJ542745–6), grey francolin (FJ542747–8), green JF (FJ542749–50), grey JF (FJ542751–2), Ceylon JF (FJ542753–4) and red JF (FJ542755–6). Bases with nucleotide A are in green, C in blue, G in yellow and T in red. Click here for file

Additional file 6 **Median-joining networks of haplotypes for all SNPs classed according to the major groups at amino acids 5 (F5L) and 520 (L520P) from Figure **3. The four possible genotypes at these positions are denoted in the legend. Branch lengths are proportional to the number of mutational differences between haplotypes. The outgroup sample branch lengths are considerably reduced in order to show the details of the chicken population network. V represents the green JF sequences; F the grey francolin; B the bamboo partridge; G the grey JF; C the Ceylon JF; R the red JF sample genotypes; and RJF the genome sequence. Click here for file

Additional file 7 **Legend to Additional file **6. Click here for file

Additional file 8 **A multiple sequence alignment of zebra finch and other bird samples protein-coding sequences**. Sites marked were candidates for selection according to PAML M8 BEB results (red), and had differences in the chicken populations compared to the red JF genome or samples (green). Regions marked with X were not resequenced. Bamboo refers to the bamboo partridge. Chicken has 2 alleles (F, L) at site 5; red JF, grey JF and bamboo partridge all have F; and Ceylon JF, green JF and grey francolin have L. At site 520 the alleles segregating in chicken (L, P) were present in chicken and red JF, and though zebra finch genome has L, the remaining birds all had P. Click here for file
