# Supplementary figures and images for: Evidence of balanced diversity at the chicken interleukin 4 receptor alpha chain locus
Source: BMC Evol Biol. 2009 Jun 15;9:136. doi: 10.1186/1471-2148-9-136 (PMC3224688; doi:10.1186/1471-2148-9-136)

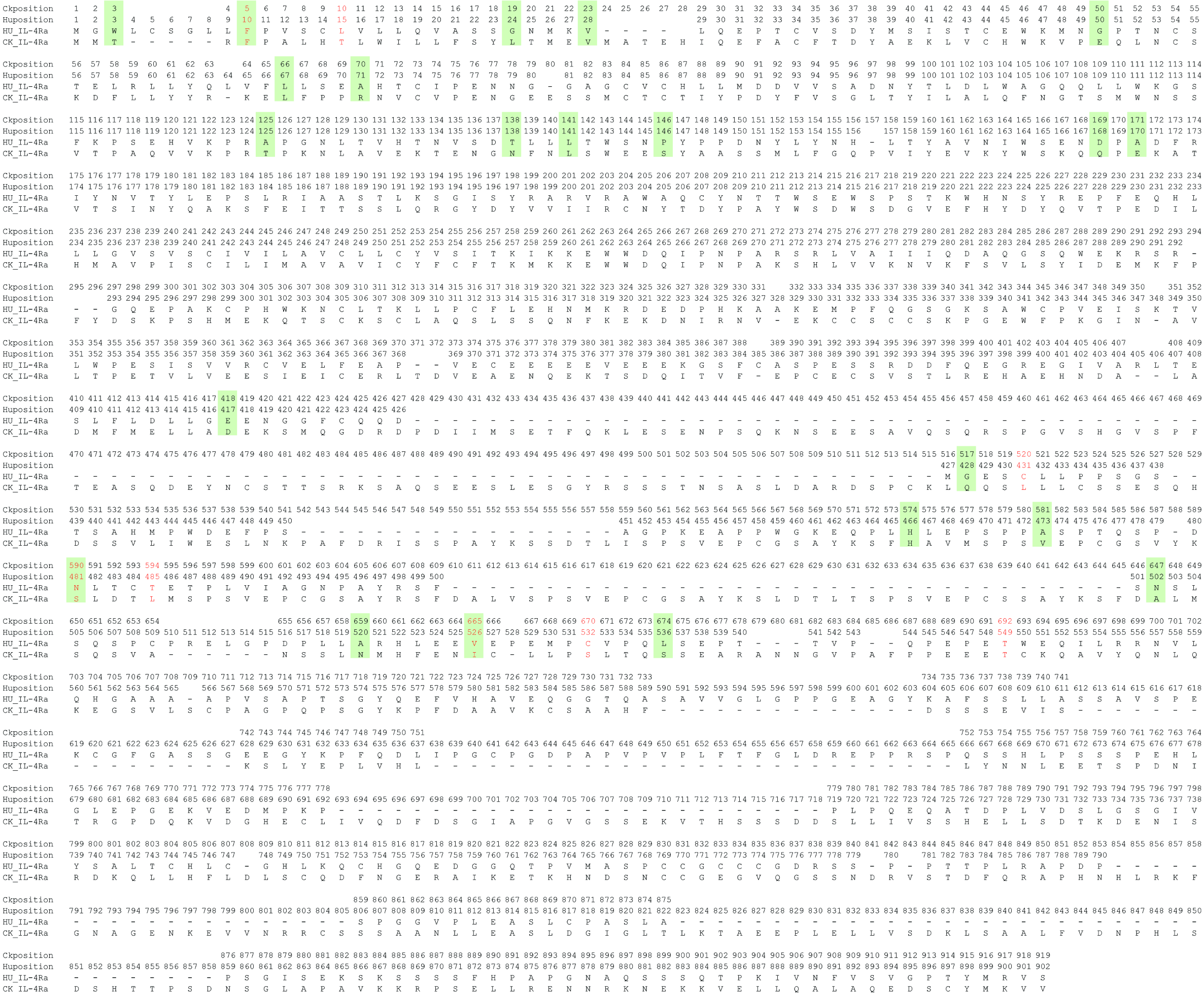

Supplement: Additional file 1 — An alignment of chicken and human IL-4Rα protein sequences. The consensus human IL-4Rα sequence isoform a (GenBank accession number NP_000409) and the consensus chicken sequence (XP_414885) were aligned with T-Coffee [12]. The sites marked green were subsequently found to be candidates for selection according to PAML M8 BEB results. Sites marked green and in red letters indicate those subsequently observed as segregating in chicken populations and/or with differences between the chicken and the red JF sequences. [file 1471-2148-9-136-S1.tiff]

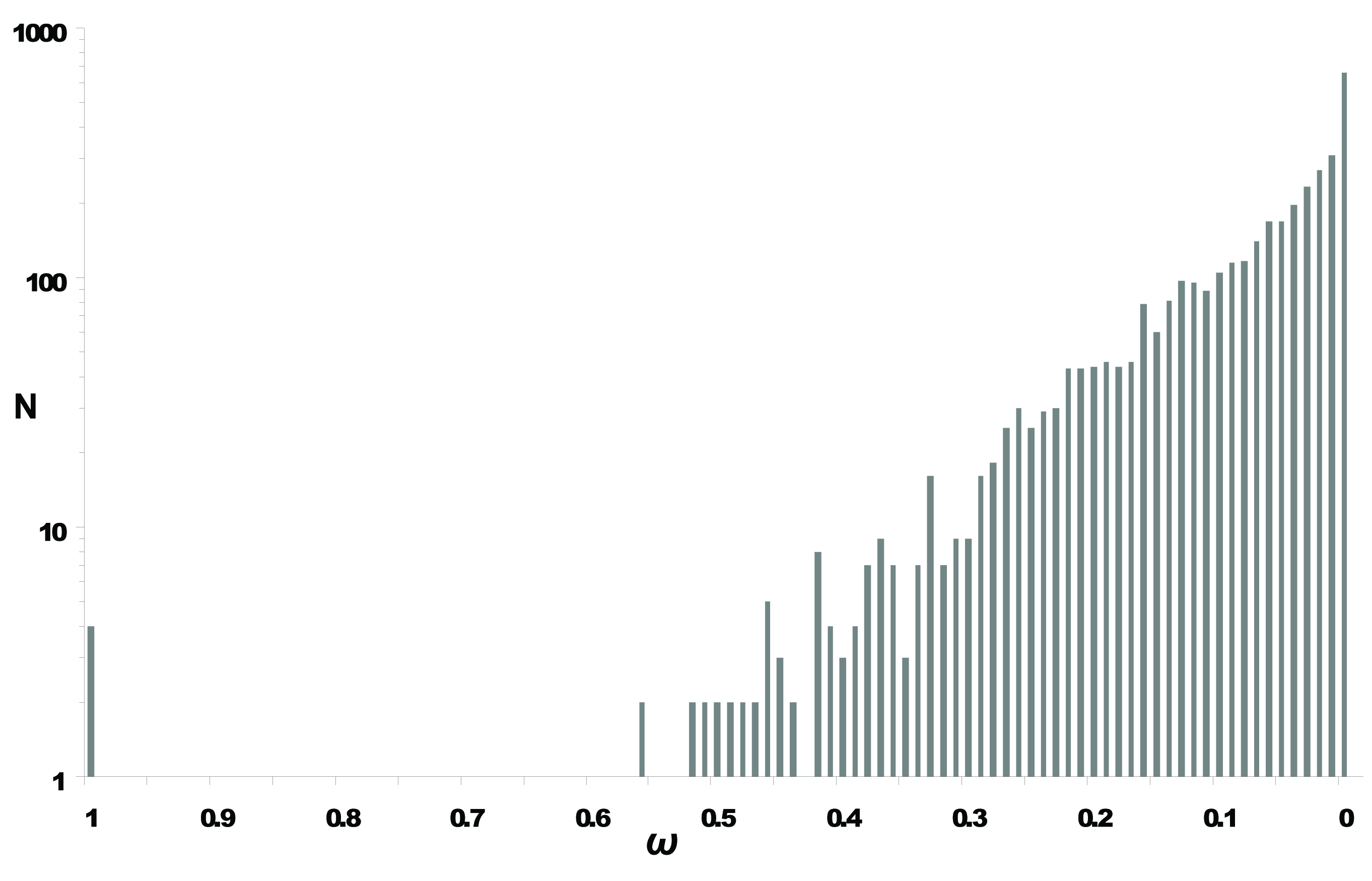

Supplement: Additional file 3 — The numbers of genes (N) in classes of ω values from pairwise alignments of chicken-zebra finch gene sets where the variable model was favoured (p < 0.05). The y-axis is on a logarithmic scale. The ω values on the x-axis are classes into groups of 0.01, with the exception of values greater than 1, which are classed as 0.99–1.00. [file 1471-2148-9-136-S3.tiff]

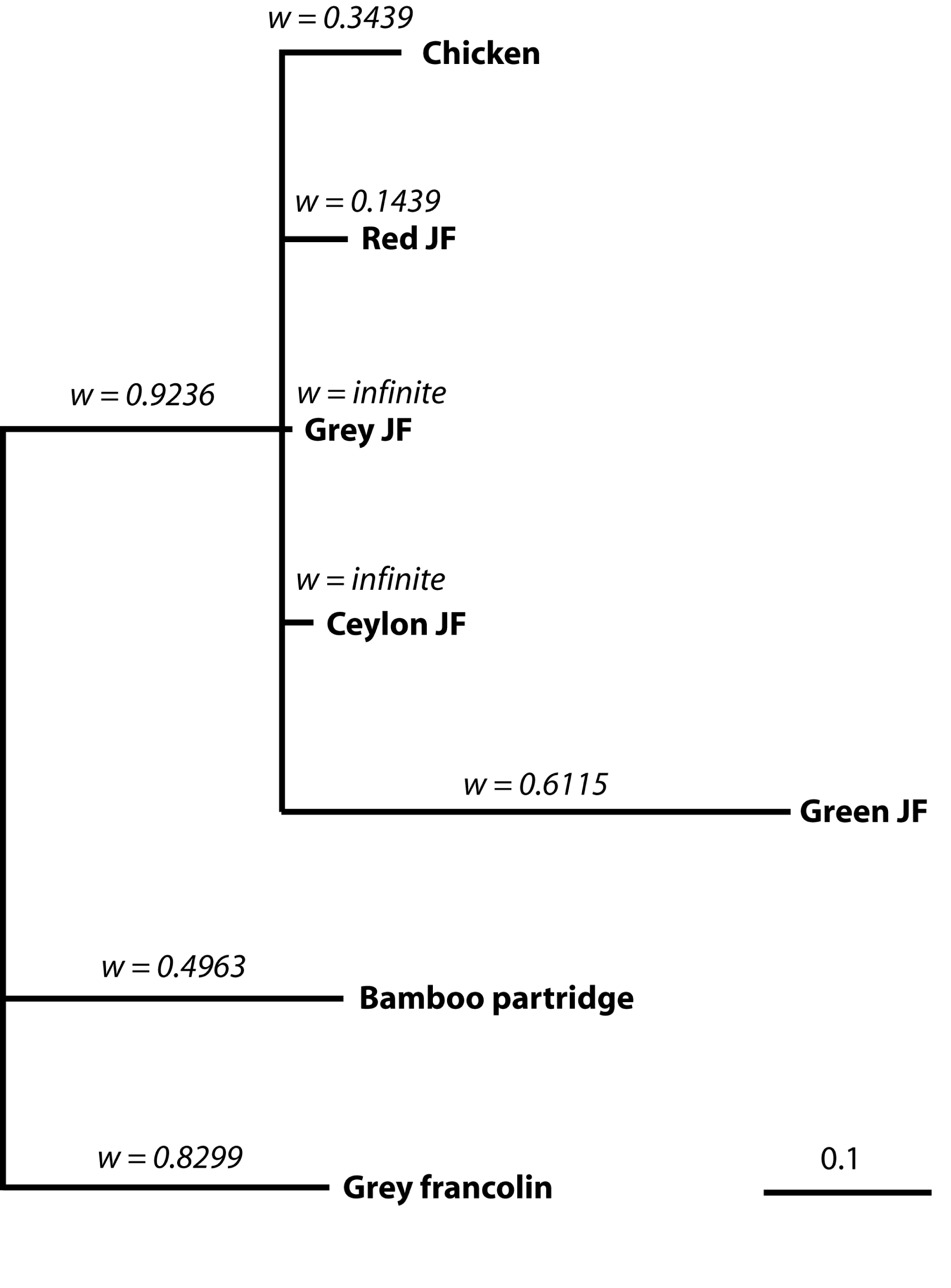

Supplement: Additional file 4 — Codeml neighbour-joining phylogeny of IL-4Rα. Branch lengths were estimated by maximum likelihood under the free-ratio model, which assumes an independent ω-ratio for each branch: these values are displayed. The branch length displayed is 0.1 of the total branch lengths for the tree. The ω for chicken was 0.4181 when sample FJ542675 was used instead of FJ542575. The ω values for grey and Ceylon JF are high because no synonymous SNPs were observed. [file 1471-2148-9-136-S4.tiff]

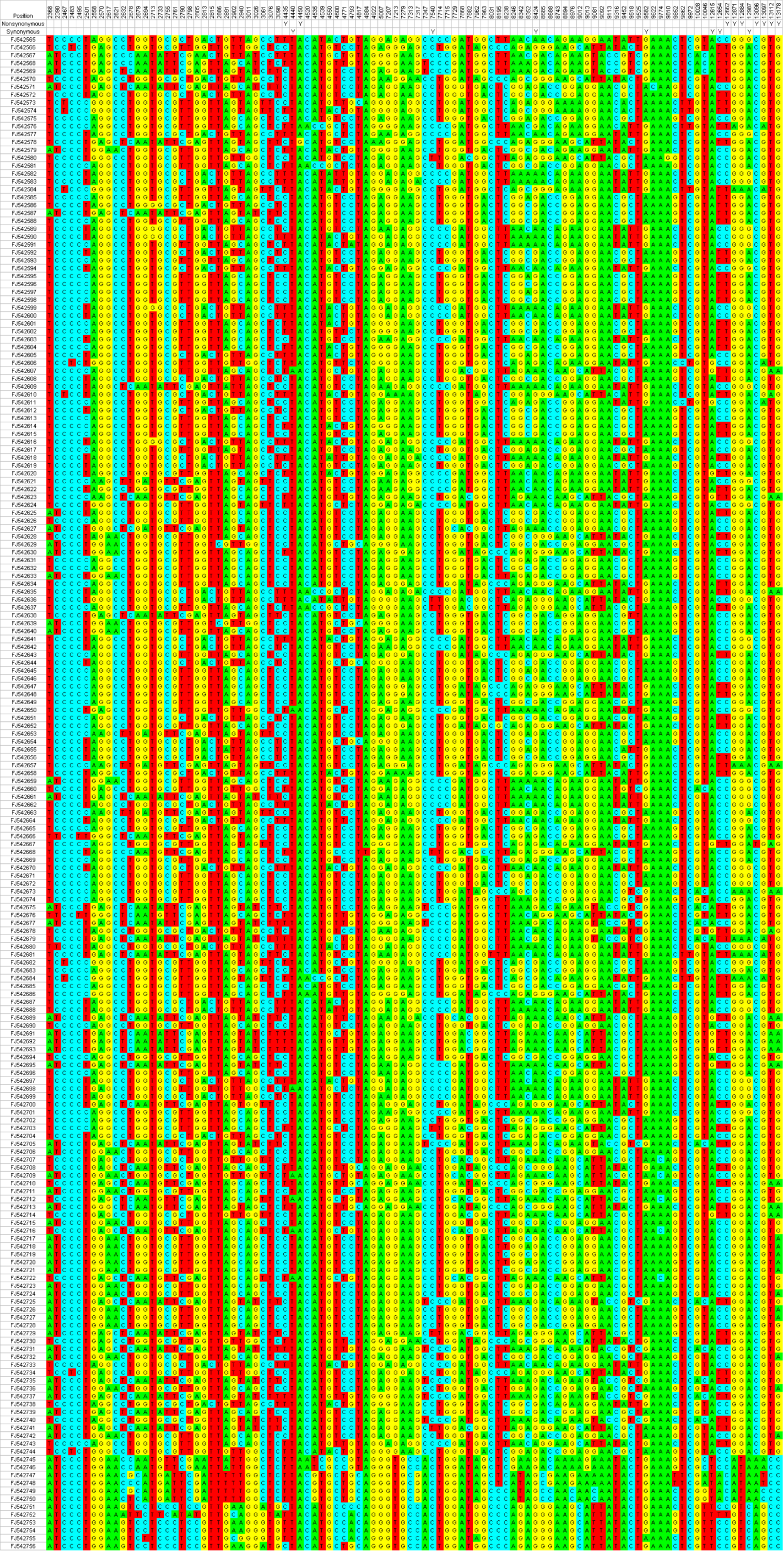

Supplement: Additional file 5 — Genotypes at SNP sites polymorphic in the chicken for all samples. The coding sites are marked as "Y" if nonsynonymous. Samples are from Pakistan (FJ542565-FJ542584), Burkina Faso (FJ542585-FJ542604), Senegal (FJ542605-FJ542624), Sri Lanka (FJ542625-FJ542644), Botswana (FJ542645-FJ542664), Bangladesh (FJ542665-FJ542684), Kenya (FJ542685-FJ542704), Broilers (FJ542705-FJ542744), bamboo partridge (FJ542745–6), grey francolin (FJ542747–8), green JF (FJ542749–50), grey JF (FJ542751–2), Ceylon JF (FJ542753–4) and red JF (FJ542755–6). Bases with nucleotide A are in green, C in blue, G in yellow and T in red. [file 1471-2148-9-136-S5.tiff]

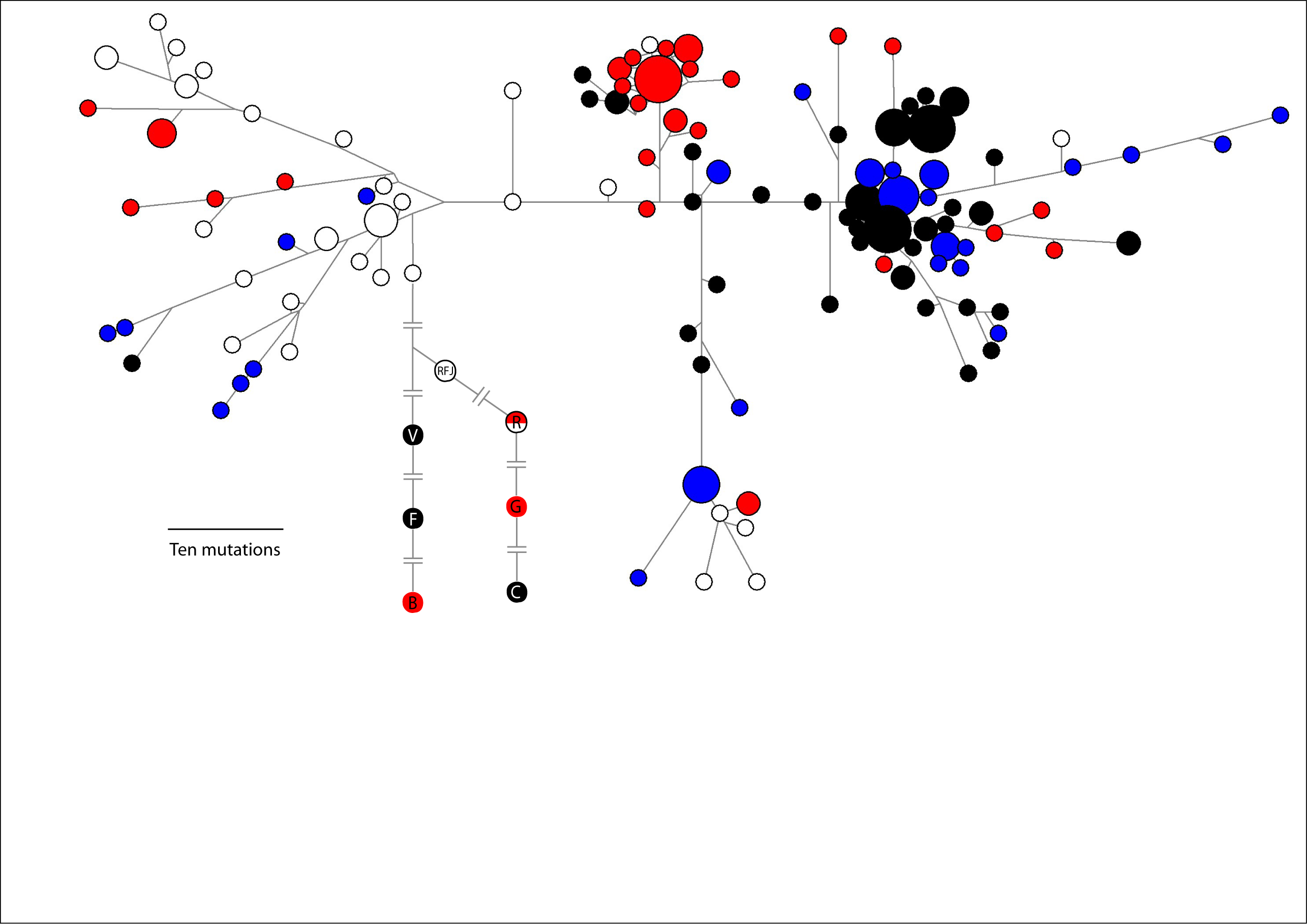

Supplement: Additional file 6 — Median-joining networks of haplotypes for all SNPs classed according to the major groups at amino acids 5 (F5L) and 520 (L520P) from Figure 3. The four possible genotypes at these positions are denoted in the legend. Branch lengths are proportional to the number of mutational differences between haplotypes. The outgroup sample branch lengths are considerably reduced in order to show the details of the chicken population network. V represents the green JF sequences; F the grey francolin; B the bamboo partridge; G the grey JF; C the Ceylon JF; R the red JF sample genotypes; and RJF the genome sequence. [file 1471-2148-9-136-S6.tiff]

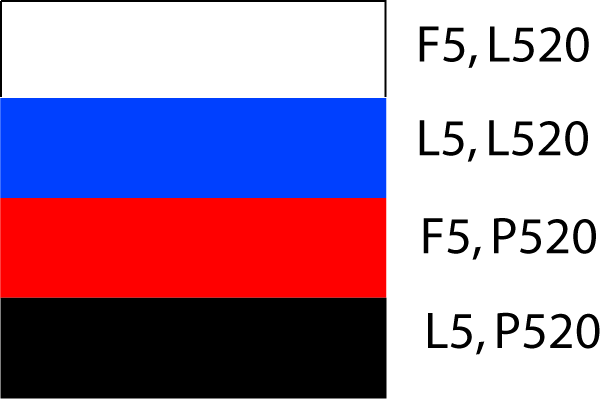

Supplement: Additional file 7 — Legend to Additional file 6. [file 1471-2148-9-136-S7.tiff]

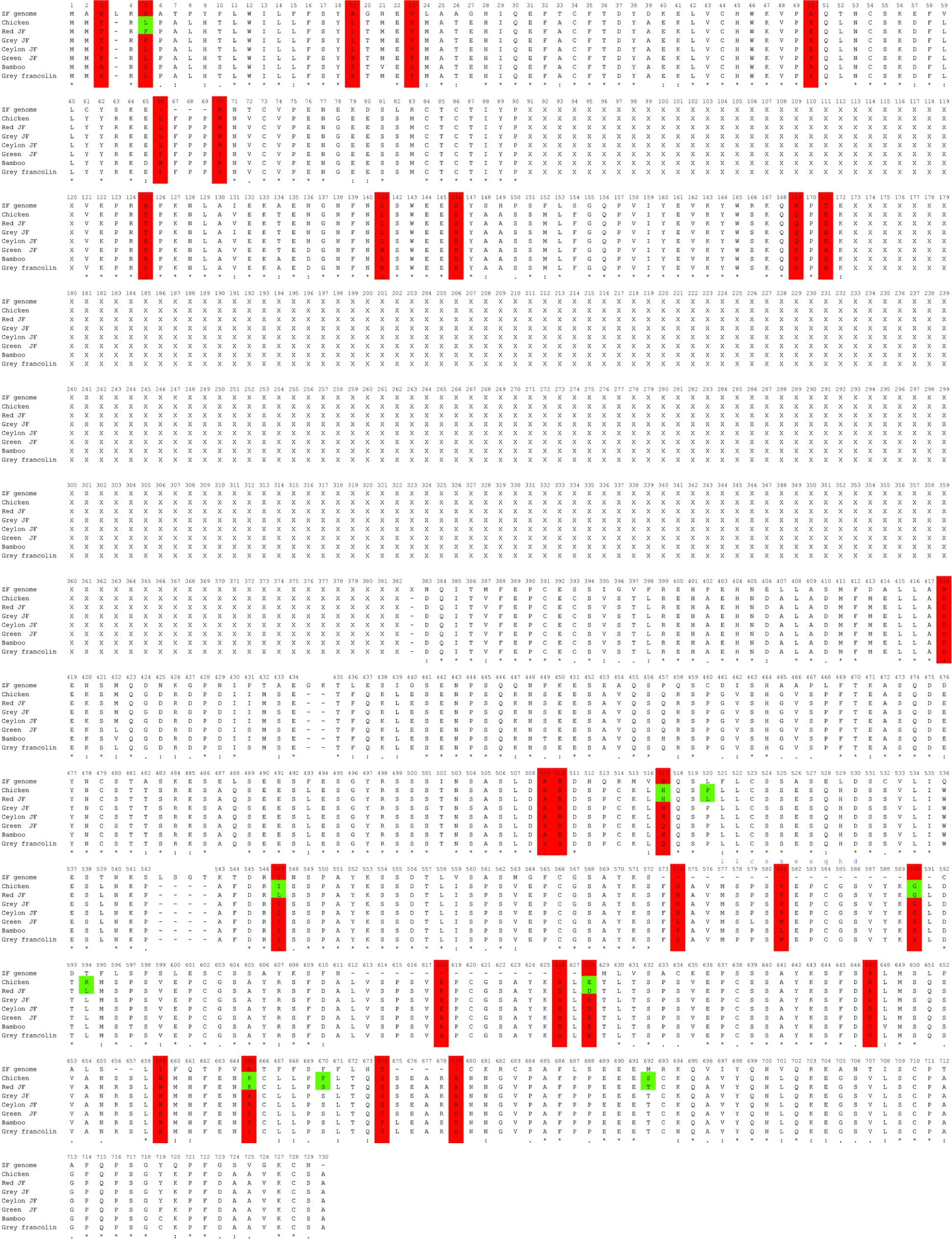

Supplement: Additional file 8 — A multiple sequence alignment of zebra finch and other bird samples protein-coding sequences. Sites marked were candidates for selection according to PAML M8 BEB results (red), and had differences in the chicken populations compared to the red JF genome or samples (green). Regions marked with X were not resequenced. Bamboo refers to the bamboo partridge. Chicken has 2 alleles (F, L) at site 5; red JF, grey JF and bamboo partridge all have F; and Ceylon JF, green JF and grey francolin have L. At site 520 the alleles segregating in chicken (L, P) were present in chicken and red JF, and though zebra finch genome has L, the remaining birds all had P. [file 1471-2148-9-136-S8.tiff]
