# Supplementary material for: Evidence of balanced diversity at the chicken interleukin 4 receptor alpha chain locus
Source: BMC Evol Biol. 2009 Jun 15;9:136. doi: 10.1186/1471-2148-9-136 (PMC3224688; doi:10.1186/1471-2148-9-136)
Supplement: Additional file 2 — Supplementary Methods and Results. A file containing details of supplementary methods and results implemented, including tables, detailing: the identification of putative chicken-zebra finch orthologous alignments, PCR and primer sequences, resequencing details, alignment parameters, methodology for pairwise comparisons of chicken and zebra finch genes, and the details of chicken genes identified that interact with IL-4Rα. [file 1471-2148-9-136-S2.doc]

**Supplementary Information**

**Supplementary Methods:**

**Identification of putative chicken-zebra finch orthologous alignments**

As described in the Methods section, the zebra finch genome was compared with the chicken genome. Chicken Refseq protein sequences (19,661) and zebra finch expressed sequence tags and mRNAs (67,671) downloaded from GenBank (www.ncbi.nlm.nih.gov) were cleaned of vector contaminants using SeqClean (http://www.tigr.org/tdb/tgi/software/), and repetitive sequences were masked using RepeatMasker (http://www.repeatmasker.org). The TIGR gene indices clustering tools (Tgicl) [10] was used to cluster the zebra finch sequences with a minimum length of 100 bases and identity of 96% for overlapping regions into 9,716 consensus contigs. These zebra finch contigs were searched against the chicken protein sequences using Blastx [11], with an E value ≤ e-10 separating best hits for each protein from paralogous sequences. The best-hit protein pairs identified in the Blastx search were aligned using T-Coffee [12]. Alignments of length < 70 amino acids and sequence identity < 60% were discarded to remove short and spurious sequences. These protein alignments were then used as templates to generate 3,653 pairwise coding sequence (CDS) alignments that were used in subsequent analyses.

**Resequencing:**

Table S1. Sets of primer pair sequences and their associated optimal PCR parameters.

| **Amplicon** | **Size (bp)** | **Orientation** | **T_M_ (^o^C)** | **[MgCl] (mM)** | **Primer Sequences** |
| --- | --- | --- | --- | --- | --- |
| 1 | 903 | Forward | 56 | 15 | ggttaggttgcaaggttttgtc |
|  |  | Reverse |  |  | ccagcccttaagatttcatgtc |
| 2 | 799 | Forward | 60 | 20 | gaatcctaacatccagcaaagc |
|  |  | Reverse |  |  | agtgaagaacacacaccaccac |
| 3 | 684 | Forward | 56 | 20 | CAGGAAAAATCCCAACTGAAAG |
|  |  | Reverse |  |  | GCACTACTTGGCAAACACTCTG |
| 4 | 708 | Forward | 61 | 15 | cagagtgtttgccaagtagtgc |
|  |  | Reverse |  |  | acatactggtgccattgaactg |
| 5 | 943 | Forward | 57 | 25 | acagttcaatggcaccagtatg |
|  |  | Reverse |  |  | ttcaggccttctcactaagctc |
| 6 | 867 | Forward | 58 | 20 | gcagtgcttgttgatgaatacc |
|  |  | Reverse |  |  | ttagatgccaactgtgttgtcc |
| 7 | 970 | Forward | 60 | 20 | AATGCAGTTTTAACCCCTGAGA |
|  |  | Reverse |  |  | GGGTTAAAGACGGTAACAAGCA |
| 8 | 906 | Forward | 62 | 20 | acaattgcagtacaaccagcag |
|  |  | Reverse |  |  | tcaaacactcatggccatctac |

Table S2. PCR cycle program used for each primer pair.

| **Step** | **Temp. (^o^C)** | **Duration** |
| --- | --- | --- |
| 1 | 95 | 15 mins |
| 2 | 95 | 30 seconds |
| 3 | T_M_ | 45 seconds |
| 4 | 72 | 60 seconds |
| 5 | 72 | 15 mins |

T_M_ is the annealing temperature as listed in Table S1. Steps 2 to 4 were repeated 33 times in sequence.

**PMut**

In some cases, the program did not have sufficient confidence in the results due to the high protein sequence divergence between chicken and other well-studied species with which it was compared. Thus the prediction outcomes were also classed as not determined in those cases.

**IL-4Rα proximity to other immune genes**

Situated on chromosome 14, the 5’ end of IL-4Rα is just 150 bp from a transcribed element (NSMCE1) [18]. The IL-21 receptor is near the 3’ end of IL-4Rα and an IL-9R precursor homolog lies close to the IL-21R as well.

**Identification of IL-4Rα in the zebra finch**

A tBlastn search [81] of the zebra finch genome sequence (July 2008 assembly) against the chicken gene protein sequence (XP_414885) and the translated versions of zebra finch sequences (DQ213788, DQ231787) identified the IL-4Rα gene on zebra finch chromosome 14. Alignments of known bird IL-4Rα gene sequences and the candidate region on chr14 using T-Coffee [12] and the tBlastn data yields a large portion of the translated zebra finch IL-4Rα coding sequence.

**The zebra finch IL-4Rα gene**

The GenBank zebra finch IL-4Rα mRNAs include 5’ UTR and perhaps a leader sequence, like the chicken copy. The zebra finch IL-4Rα coding region starts with exon 1 at base chr14:16,260,749 is 69 bases long. Other identifiable orthologous coding regions to chicken are exon 2 at 16,264,259-402, exon 3 at 16,265,272-427, exon 4 at 16,266,443-604, exon 5 at 16,267,141-299, exon 8 at 16,268,959-9,036 and exon 9 at 16,269,132-71,277. Regions for exons 6, 7 and 10 were not clear as the zebra finch mRNA sequences are short and the divergence between chicken and zebra finch is high at the 3’ end of the gene, which is reflected in the number of segregating polymorphism in the chicken samples.

An alignment of IL-4Rα protein sequences from the zebra finch and other bird sequences using T-Coffee [12] was examined for substitutions among species.

[81] Altschul SF, Gish W, Miller W, Myers EW, Lipman DJ: **Basic local alignment search tool.**  *J. Mol. Biol.* 1990, **215**:403-410.

**Supplementary Results:**

**IL-4Rα Codeml Alignment Parameters**

The IL-4Rα mRNA sequence (XM_414885) aligned as a best hit to 2 clustered zebra finch mRNAs with a Blastx score of 339 and an e-value of 2e-92. A LRT of the variable and fixed model pairwise comparisons showed that the variable model has a log-likelihood of -1422.79 with *ω* = 0.5098. The neutral model log-likelihood with *ω* = 1 was -1427.74, so the variable model was significantly more likely with p = 0.0017.

**Pairwise comparisons of chicken and zebra finch genes:**

A set of 3,653 chicken and zebra finch CDS pairwise alignments were examined to identify candidate genes potentially subject to directional selection. Ten candidate genes were observed with ω > 0.5 where the variable model was significantly favoured. The most represented functional category among the 10 candidate genes was related to immunity. Three genes have roles in the immune response: IL-4Rα, protein inhibitor of activated STAT 2 (Pias2) and progesterone-induced blocking factor 1 (Pibf). Other functional categories included apoptosis (G-2 and S-phase expressed 1), signalling (a phosphatase and an anion exchanger), and intracellular structure (NADH DH 1β 6 and GORASP2). Functions for two genes were unknown. Two of the genes with ω > 1 were not valid coding sequences; the other two were a phosphatase (PPM1K) and an unannotated sequence.

**Chicken genes identified that interact with IL-4Rα:**

Interestingly, the two other chicken immune genes identified by this pairwise comparison method (Pibf and Pias2) have human orthologs that interact with each another and IL-4Rα. Human Pibf is an immunoregulatory factor expressed during embryo development that regulates T_H_1 and T_H_2 cytokine production balance by binding the IL-4Rα and an anchored Pibf receptor chain, which activates Janus kinase 1 (Jak1) to phosphorylate STAT6 [60]. Normally, activated STAT6 proteins dimerise and translocate to the nucleus, where they activate T_H_2 cytokines [61-62]. However, human Pias proteins may prevent cytokine activation by inhibiting STAT proteins in the nucleus [63-64]. Thus the 3 immune genes identified with this method not only are expected to interact in the same pathway but also are likely to have crucial roles in modulating the immune response of chickens to viruses, bacteria and parasites.

Table S3. Pairwise comparison details and functions for chicken sequences with *ω* > 0.5 and p < 0.05.

| CK refseq | ZF Contig | *ω*=*d_N_*/*d_S_* | 2*ΔML* | P value | *d_N_* | *d_S_* | Chicken gene name | ZF contig information | Human Function | Chicken GenBank description |
| --- | --- | --- | --- | --- | --- | --- | --- | --- | --- | --- |
| XM_420574 | CL1281Contig1 | 3.0968 | 5.668 | 0.0173 | 3.7408 | 1.2080 | LOC422614 | Phosphatase | Signalling | Protein phosphatase 1K (PP2C domain containing) |
| XM_414705 | CL522Contig1 | 0.5373 | 4.334 | 0.0374 | 0.2246 | 0.4180 | NDUFB6 | NADH DH 1β | Structure | NADH dehydrogenase (ubiquinone) 1 beta subcomplex, 6, 17kDa |
| XM_419473 | CL560Contig1 | 0.5162 | 4.776 | 0.0289 | 0.2010 | 0.3895 | SLC4A1AP | Kanadaptin | Signalling | Solute carrier family 4 (anion exchanger), member 1, adaptor |
| NM_001012594 | CL6285Contig1 | 0.5127 | 4.430 | 0.0353 | 0.1569 | 0.3061 | GORASP2 | - | Structure | Golgi reassembly stacking protein 2, 55kDa |
| NM_001031332 | CL4994Contig1 | 0.5511 | 8.708 | 0.0032 | 0.2542 | 0.4612 | GTSE1 | - | Apoptosis | G-2 and S-phase expressed 1 |
| XM_414885 * | CL6154Contig1 | 0.5098 | 9.896 | 0.0017 | 0.1791 | 0.3514 | LOC416585 | IL-4R α-chain | Immunity | Interleukin-4 receptor alpha-chain |
| NM_001030626 | CL4084Contig1 | 0.5665 | 6.796 | 0.0091 | 0.4255 | 0.7511 | PIAS2 | - | Immunity | Protein inhibitor of activated STAT, 2 |
| XM_417014 | CL4220Contig1 | 0.5666 | 4.006 | 0.0453 | 0.1145 | 0.2021 | LOC418820 | - | Immunity | Progesterone-induced blocking factor 1 |
| XM_420836 | CL4938Contig1 | 4.0730 | 6.172 | 0.0130 | 4.0265 | 0.9886 | LOC422894 | - | - | - |
| XM_001234647 | CL3015Contig1 | 0.5215 | 3.988 | 0.0458 | 0.1965 | 0.3768 | LOC771361 | Gal-4NAc-4-sulfotr | - | - |
|  | CL2548Contig1 | 0.5082 | 7.616 | 0.0058 | 0.5438 | 1.0701 |  |  |  |  |

* IL-4Rα, the chicken gene selected for resequencing. 2*ΔML* is twice the difference of the maximum likelihood values of the variable model minus the fixed model.

Table S4. Estimated distribution of synonymous (S.d_S_) and nonsynonymous (N.d_N_) SNPs by the codeml free-ratio model.

| Sample | N.d_N_ | S.d_S_ |
| --- | --- | --- |
| Chicken ^1^ | 5.9 | 5.5 |
| Red JF ^1^ | 2.0 | 4.4 |
| Grey JF ^1^ | 1.0 | 0 |
| Ceylon JF ^1^ | 3.0 | 0 |
| Green JF ^1^ | 32.2 | 16.8 |
| Bamboo partridge | 20.0 | 12.9 |
| Grey francolin | 22.7 | 8.7 |

^1^ On the branch ancestral to the *Gallus* birds, 19.8 nonsynonymous and 6.8 synonymous mutations were observed.

Additional file 1. An alignment of chicken and human IL-4Rα protein sequences.


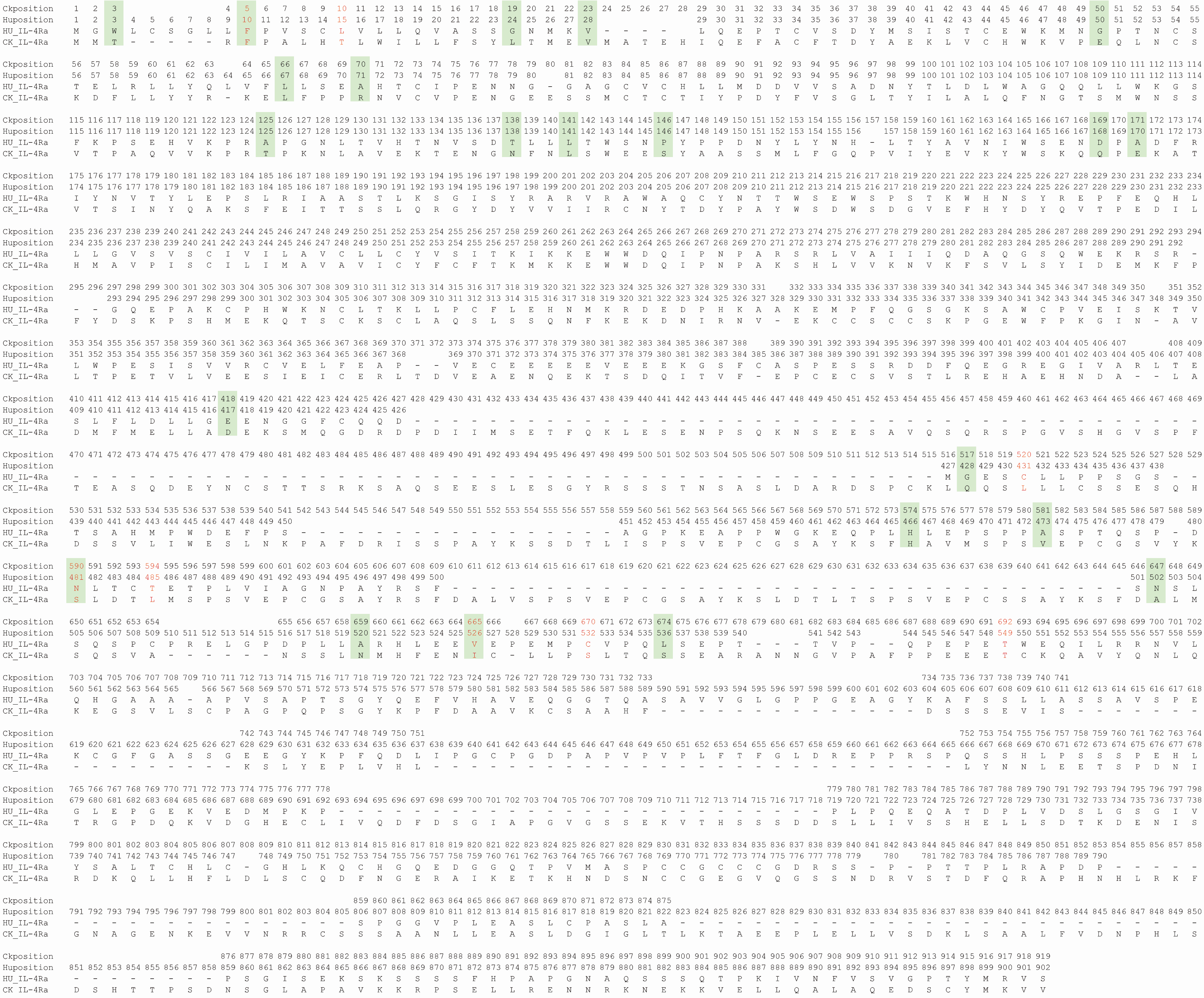


The consensus human IL-4Rα sequence isoform a (GenBank accession number NP_000409) and the consensus chicken sequence (XP_414885) were aligned with T-Coffee [12]. The sites marked green were subsequently found to be candidates for selection according to PAML M8 BEB results. Sites marked green and in red letters indicate those subsequently observed as segregating in chicken populations and/or with differences between the chicken and the red JF sequences.

Additional file 3. The numbers of genes (N) in classes of ω values from pairwise alignments of chicken-zebra finch gene sets where the variable model was favoured (p<0.05).

The y-axis is on a logarithmic scale. The ω values on the x-axis are classes into groups of 0.01, with the exception of values greater than 1, which are classed as 0.99-1.00.

Additional file 4. Codeml neighbour-joining phylogeny of IL-4Rα.


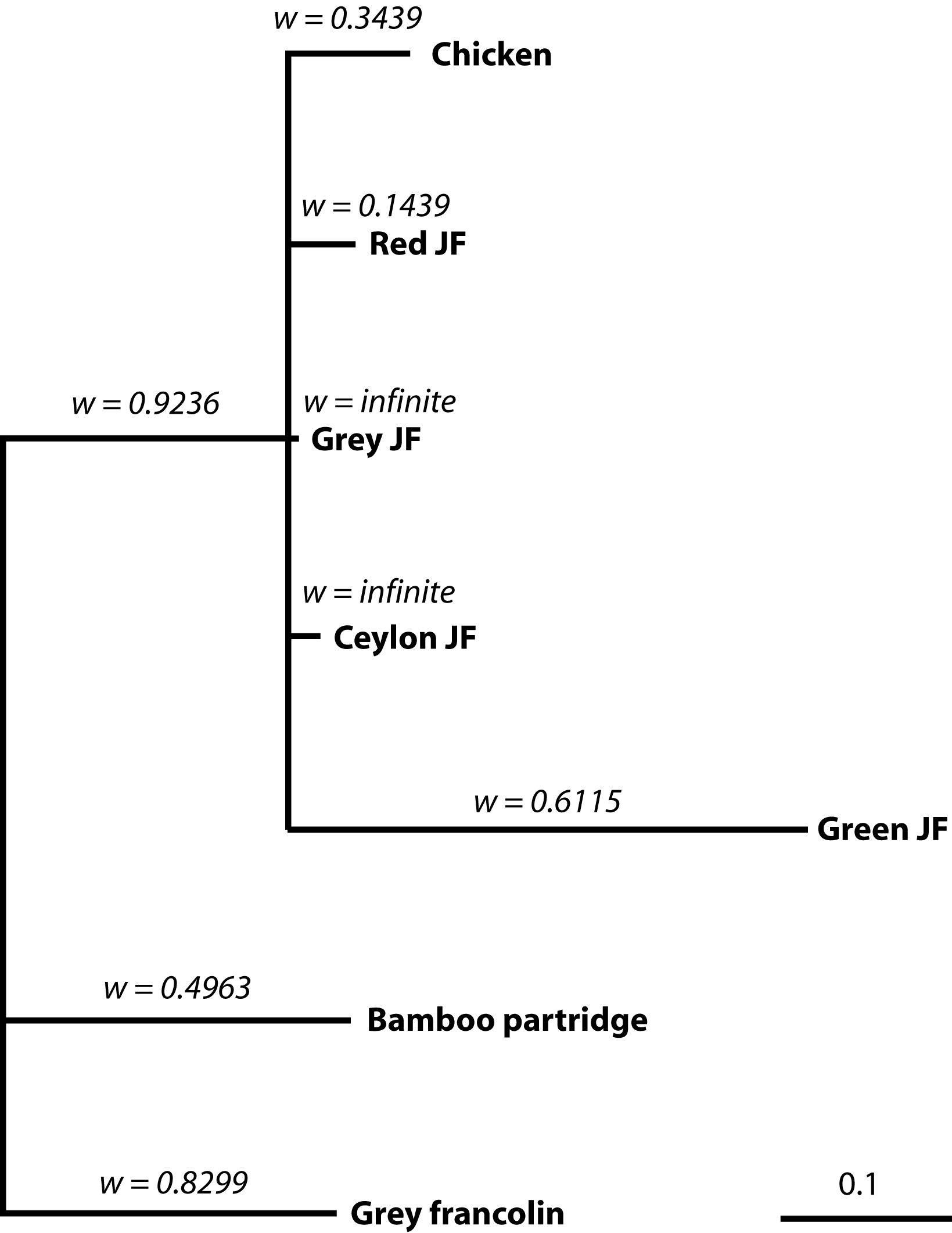


Branch lengths were estimated by maximum likelihood under the free-ratio model, which assumes an independent *ω*-ratio for each branch: these values are displayed. The branch length displayed is 0.1 of the total branch lengths for the tree. The *ω* for chicken was 0.4181 when sample FJ542675 was used instead of FJ542575. The *ω* values for grey and Ceylon JF are high because no synonymous SNPs were observed.

Table S5. Protein function impacts predicted by PMut for candidate M8 BEB sites under selection and polymorphic between the chicken and the red JF genome sequence.

| Base  Position | Amino Acid | | | Prediction | Score | Certainty | Outcome |
| --- | --- | --- | --- | --- | --- | --- | --- |
|  | Position | Red JF | Alt. |  |  |  |  |
| 4435-37 | 5 | F | L | neutral | 0.316 | 3 | not significant |
| 12652-54 | 517 | Q | H ^3^ | neutral | 0.095 | 8 | neutral |
| 12742-44 | 547 ^1^ | L | I | neutral | 0.026 | 9 | neutral |
| 12871-73 | 590 | S | G | neutral | 0.329 | 3 | not significant |
| 12985-87 | 628 ^2^ | D | E | neutral | 0.035 | 9 | neutral |
| 13096-98 | 665 | M | R | neutral | 0.495 | 0 | not significant |
| 13096-98 | 665 | M | Q ^4^ | neutral | 0.119 | 7 | neutral |
| 13096-98 | 665 ^1^ | R | Q ^4^ | neutral | 0.510 | 5 | not significant |

Alt. stands for alternative allele. ^1^ Polymorphic between the chicken and the red JF sample. ^2^ The red JF allele was the same for the genome sequence and sample. ^3^ The red JF sample and some chickens shared a synonymous SNP at this site. ^4^ The chicken minor allele at the site. Substitutions where the PMut certainty values ≤ 6 did not have statistical support for the predicted change.

Additional file 5. Genotypes at SNP sites polymorphic in the chicken for all samples.





The coding sites are marked as “Y” if nonsynonymous. Samples are from Pakistan (FJ542565-FJ542584), Burkina Faso (FJ542585-FJ542604), Senegal (FJ542605-FJ542624), Sri Lanka (FJ542625-FJ542644), Botswana (FJ542645-FJ542664), Bangladesh (FJ542665-FJ542684), Kenya (FJ542685-FJ542704), Broilers (FJ542705-FJ542744), bamboo partridge (FJ542745-6), grey francolin (FJ542747-8), green JF (FJ542749-50), grey JF (FJ542751-2), Ceylon JF (FJ542753-4) and red JF (FJ542755-6). Bases with nucleotide A are in green, C in blue, G in yellow and T in red.

Table S6. Tajima’s *D* and Fay and Wu’s *H* for each Asian and African population.

For each population, 10 chickens were sampled each.

| Continent | Asia | | | Africa | | | |
| --- | --- | --- | --- | --- | --- | --- | --- |
| Population | Bangladesh | Pakistan | Sri Lanka | Botswana | Burkina Faso | Senegal | Kenya |
| SNPs | 82 | 86 | 73 | 74 | 51 | 70 | 71 |
| Tajima’s *D* | 0.85 | 1.10 | 0.86 | 0.90 | 1.21 | 1.03 | 1.81 |
| *P value* | *0.032* | *0.006* | *0.033* | *0.015* | *0.004* | *0.012* | *<0.001* |
| Fay & Wu’s *H* | -14.33 | -17.58 | -22.23 | -22.32 | -21.85 | -14.22 | -12.41 |
| *P value* | *0.031* | *0.027* | *0.004* | *0.003* | *<0.001* | *0.016* | *0.037* |

Additional file 6. Median-joining networks of haplotypes for all SNPs classed according to the major groups at amino acids 5 (F5L) and 520 (L520P) from Figure 2b.


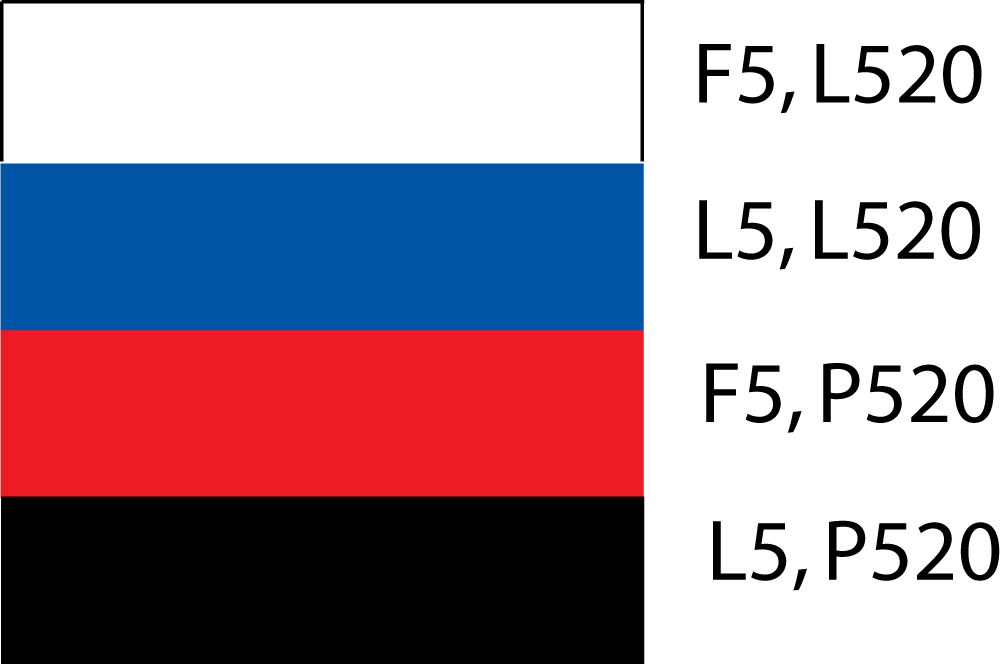

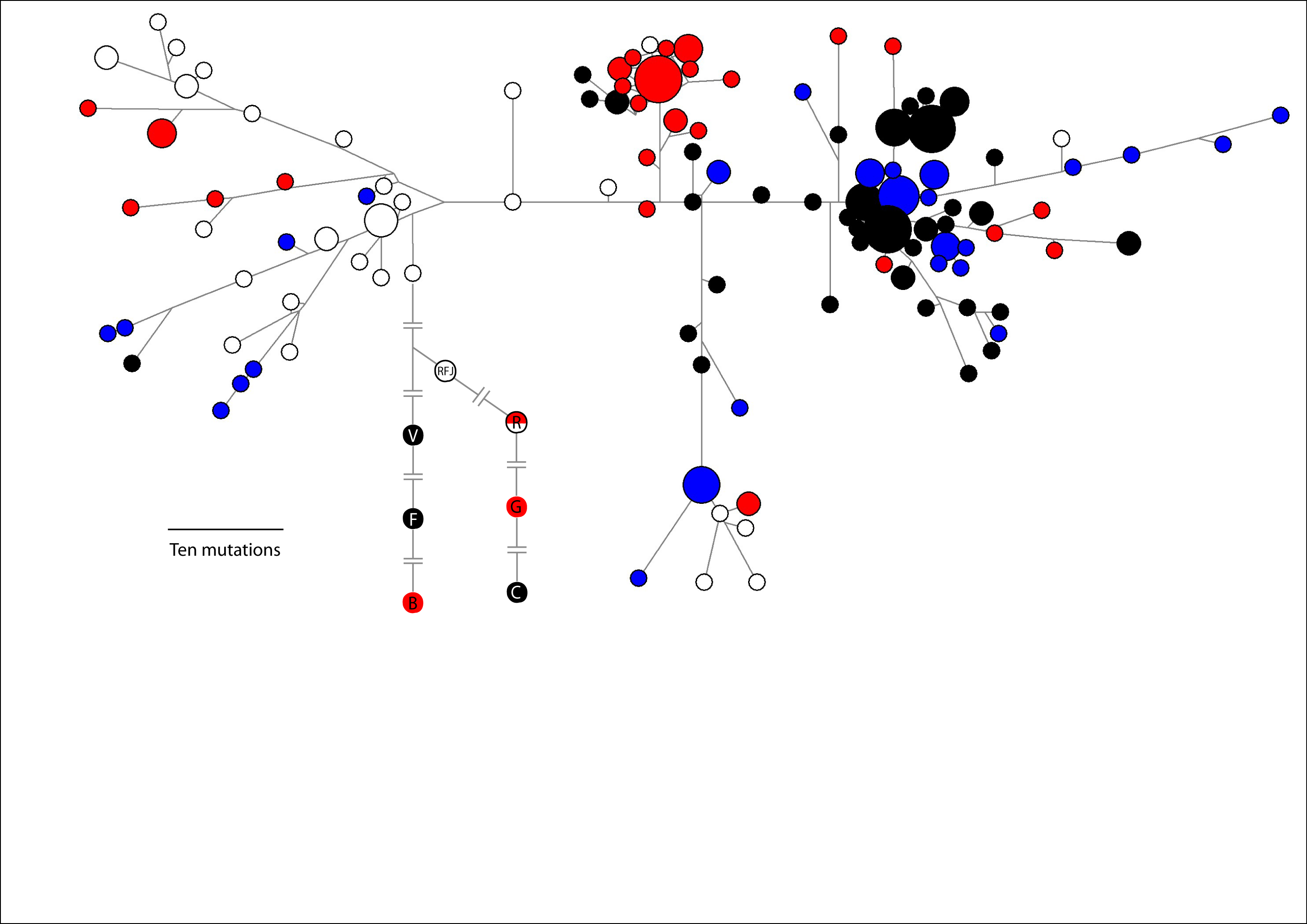


The four possible genotypes at these positions are denoted in the legend. Branch lengths are proportional to the number of mutational differences between haplotypes. The outgroup sample branch lengths are considerably reduced in order to show the details of the chicken population network. V represents the green JF sequences; F the grey francolin; B the bamboo partridge; G the grey JF; C the Ceylon JF; R the red JF sample genotypes; and RJF the genome sequence.

Additional file 8. A multiple sequence alignment of zebra finch and other bird samples protein-coding sequences.


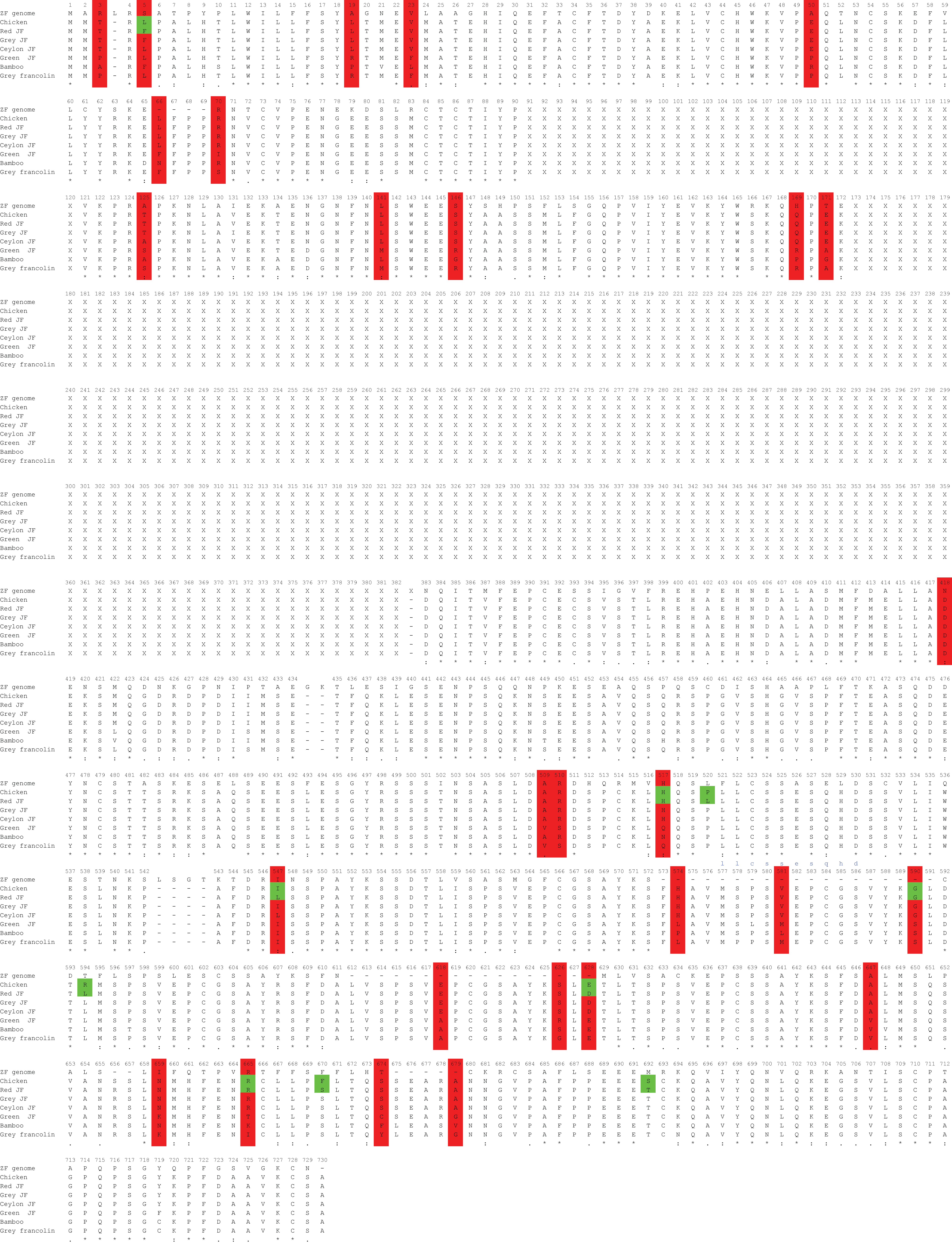


Sites marked were candidates for selection according to PAML M8 BEB results (red), and had differences in the chicken populations compared to the red JF genome or samples (green). Regions marked with X were not resequenced. Bamboo refers to the bamboo partridge. Chicken has 2 alleles (F, L) at site 5; red JF, grey JF and bamboo partridge all have F; and Ceylon JF, green JF and grey francolin have L. At site 520 the alleles segregating in chicken (L, P) were present in chicken and red JF, and though zebra finch genome has L, the remaining birds all had P.
